# Skin Barrier Dysregulation in Psoriasis

**DOI:** 10.3390/ijms221910841

**Published:** 2021-10-07

**Authors:** Andreas Orsmond, Lara Bereza-Malcolm, Tom Lynch, Lyn March, Meilang Xue

**Affiliations:** 1 Sutton Arthritis Research Laboratory, Faculty of Medicine and Health, Institute of Bone and Joint Research, Kolling Institute, University of Sydney at Royal North Shore Hospital, St Leonards, NSW 2065, Australia; aors8205@uni.sydney.edu.au (A.O.); lara.bereza-malcolm@sydney.edu.au (L.B.-M.); 2 The Australian Arthritis and Autoimmune Biobank Collaborative (A3BC), Faculty of Medicine and Health, Institute of Bone and Joint Research, Kolling Institute, University of Sydney at Royal North Shore Hospital, St Leonards, NSW 2065, Australia; tom.lynch@sydney.edu.au (T.L.); lyn.march@sydney.edu.au (L.M.)

**Keywords:** skin barrier, psoriasis, keratinocyte, immune cells, genetic aberration, environmental factors, treatment

## Abstract

The skin barrier is broadly composed of two elements—a physical barrier mostly localised in the epidermis, and an immune barrier localised in both the dermis and epidermis. These two systems interact cooperatively to maintain skin homeostasis and overall human health. However, if dysregulated, several skin diseases may arise. Psoriasis is one of the most prevalent skin diseases associated with disrupted barrier function. It is characterised by the formation of psoriatic lesions, the aberrant differentiation and proliferation of keratinocytes, and excessive inflammation. In this review, we summarize recent discoveries in disease pathogenesis, including the contribution of keratinocytes, immune cells, genetic and environmental factors, and how they advance current and future treatments.

## 1. Introduction

**Figure 1:**
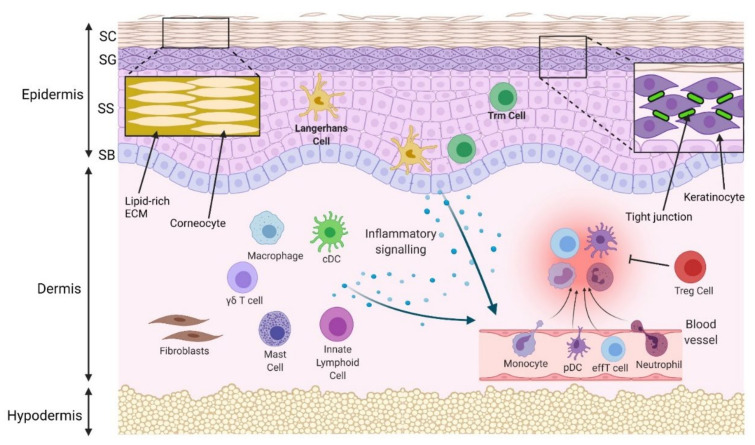
Physical and immune skin barrier in normal conditions. Densely packed corneocytes, surrounded by a lipidrich extracellular matrix (ECM) in the stratum corneum (SC), prevent passage of water and pathogens. Tight junctions between keratinocytes in the stratum granulosum (SG) prevent paracellular molecular passage. The stratum spinosum (SS) and stratum basale (SB) are deeper and are the initiation site of programmed keratinocyte proliferation and differentiation. Langerhans cells and some T resident memory (Trm) cells are located in the epidermis, while macrophages, conventional dendritic cells (cDCs), mast cells, innate lymphoid cells, γδ T cells and regulatory T cells (Tregs) are located in the uninflamed dermis. In response to pathogens, injury or allergens, these cells are activated. A number of different cytokines and chemokines may be released to compel the infiltration of inflammatory cells, such as neutrophils, monocytes, plasmacytoid dendritic cells (pDCs) and effector T cells (effT) from blood vessels. Collectively, they remove the invaded pathogens and clear cell debris. Regulatory cells resolve inflammation, and the skin barrier is maintained.

The skin is the largest organ of the human body, and consists of three layers—the epidermis, dermis, and hypodermis. The epidermis, the most superficial structure, is comprised of specialized cells called keratinocytes that form four distinct epidermal layers—the stratum basale, stratum spinosum, stratum granulosum and stratum corneum. The dermis resides below the epidermis and largely consists of a fibrous extracellular matrix (ECM) generated by local fibroblasts with interspersed resident immune cells. The hypodermis is the deepest layer and is mostly fatty tissue. These structures combine to form an effective barrier (Figure 1) that protects the body from external environmental insults through physical and immune system functions [1].

The skin’s physical barrier function is mainly carried out by the epidermis. The stratum corneum prevents structural damage and the passage of moisture and pathogens due to its dense composition of differentiated keratinocytes (corneocytes) surrounded by a lipidrich ECM [2]. The tight junctions (TJs) that connect keratinocytes in the stratum granulosum also facilitate barrier function by preventing the paracellular passage of water, nutrients, pathogens, and harmful chemicals. TJ proteins consist of a complex network of intra and extracellular proteins, including occludin, cingulin, zonula occludens (ZO), claudins and junctional adhesion molecule-A [3]. They can become dysregulated in response to radiation, infection, and inflammatory processes [4].

The functions of the immune barrier are predominantly carried out by specialised immune cells in the skin (Figure 1). The most superficial immune cells are Langerhans cells (LCs), a subtype of dendritic cells (DCs), which reside in the epidermis and have antigen-presenting functions [5]. Other DC subtypes are found in the dermis. Conventional (c)DCs are present in the steady state and also infiltrate during inflammation, whereas plasmacytoid (p)DCs are rarely found in healthy skin, and only migrate into the dermis after receiving inflammatory signals [6]. Neutrophils, monocytes and T cells, additionally, migrate into the dermis following inflammatory signalling [7]. In the steady state, skin T cells are largely resident memory T cells (T_RM_), which include epidermal T_RM_ CD8+ cells and dermal T_RM_ CD4+ cells [8]. Other skin resident immune cells include innate lymphoid cells (ILCs), mast cells, and γδ T cells [8], which are crucial in initiating inflammatory cytokine responses. Traditional CD4+ regulatory T cells and the newly studied tissue resident memory regulatory T cells also both localise in the skin and resolve immune responses [9]. Keratinocytes, additionally, facilitate the immune barrier by releasing antimicrobial molecules (notably, β-defensins and cathelicidins) and cytokines that activate dermal leucocytes [7]. 

The physical and immune skin barriers form a cooperative network to maintain skin homeostasis. However, dysregulation can contribute to many inflammatory skin disorders, including psoriasis. 

## 2. Psoriasis 

Psoriasis is a skin disease that develops following chronic inflammatory signalling and keratinocyte hyperproliferation [10,11]. It is associated with substantial physical and psychological disability that stems from the pain of the skin lesions, poor body image and extensive comorbidities [12,13]. Recent estimates of global psoriasis prevalence range between 30-65 million people [14,15], with substantial variation between nationalities [16]. 

Psoriasis is commonly classified by several factors, including age of onset, severity, and anatomical site (e.g., nail, scalp, genital) [17]. The disease can also be categorised into several clinical variants, including plaque, guttate, erythrodermic and pustular psoriasis [11]. Plaque psoriasis is characterised by scaly plaques which concentrate in the elbows, knees, and scalp. Guttate psoriasis typically features smaller lesions on the trunk, and often follows streptococcal infections. Erythrodermic psoriasis is a variant that occurs when psoriatic skin lesions cover the majority of the body, which can be life threatening if untreated. Finally, pustular psoriasis features painful and purulent skin lesions, which can be general or localised [17]. Chronic plaque psoriasis is the most studied phenotype, as it accounts for 90% of cases [17,18]. Unless otherwise specified, chronic plaque psoriasis will be the focus of this review.

Psoriasis was initially believed to be a primary disorder of excessive keratinocyte proliferation. However, successful immunomodulatory treatments and insights into the pathological mechanisms of psoriasis have shown that psoriasis is primarily initiated by immune signalling characterised by T-cell hyperactivity [19]. In this review, we detail how the breach of physical and immune skin barriers contributes to psoriasis pathogenesis and explain how these processes relate to current and future treatments. 

## 3. Barrier Aberration in Psoriasis 

### 3.1. Physical Barrier Disruption

In psoriasis, the epidermal physical barrier becomes dysregulated. The hyperproliferation and abnormal differentiation of keratinocytes lead to the development of skin lesions, which results in epidermal structural damage and barrier dysfunction.

#### 3.1.1. Disruption of Keratinocyte Proliferation and Differentiation

Psoriatic plaques feature hyperkeratosis (the thickening of the stratum corneum) and acanthosis (the thickening of other epidermal layers), resulting from uninhibited proliferation and the abnormal differentiation of keratinocytes [19]. Specifically, plaques have a cell density that is 2-5 times higher and a transit time through programmed differentiation that is 5-7 times faster than normal skin [20]. Correspondingly, epidermal proliferation markers—including Ki-67, cyclin D1 and cyclin E, retinoblastoma protein, proliferating cell nuclear antigen and p63—are highly expressed in lesions, and are reduced following effective treatment [21,22].

Markers of keratinocyte differentiation are also aberrantly expressed in psoriatic lesions. Keratins are the primary intermediate filaments that support keratinocyte structure, and their expression reflects the differentiation state of keratinocytes. Keratin 14-5 and 10-1 pairs mediate typical keratinocyte expansion and terminal differentiation, and have tightly regulated expressions in the basal and suprabasal epidermal layers, respectively [23]. However, in psoriatic lesions, keratin 14 is expressed throughout the epidermis, and is atypically coexpressed with keratin 10 in some suprabasal keratinocytes [24]. Similarly, a hallmark of psoriasis is keratinocyte expression of keratin 6, 16 and 17, which are normally not expressed in the epidermis [23]. This may be particularly important as therapies targeting keratin 17 mediated signalling have achieved promising outcomes in psoriasislike mouse models [25]. Mutations in keratins 10, 14, 16 and 17 are associated with psoriasis [26]. The epidermal differentiation markers loricrin and filaggrin also have reduced expressions in psoriatic lesions [27,28]. The corresponding disruption of programmed keratinocyte differentiation, additionally, leads to a markedly thin or absent stratum granulosum and nucleated corneocytes in skin lesions [29].

Keratinocytes primarily proliferate excessively in response to psoriatic inflammatory signalling from the skin immune system. Interleukin (IL)-17 is the main contributor [30]. IL-17 may promote proliferation by activating the nuclear factor-κB activator 1 protein downstream of the IL-17 receptor, or by activating the epidermal growth factor receptor [31,32]. IL-22 is also noteworthy, because it compels keratinocytes to replicate a tumourlike phenotype, featuring enhanced mitogen activated protein kinase signalling, the inhibition of apoptosis, and increased markers of stemness [33,34,35].

Interestingly, not all signalling from keratinocytes in psoriasis is proproliferative. Skin lesions have high expressions of transglutaminase (TG) 1 and 3 [36], which facilitate programmed keratinocyte differentiation and prevent the development of aberrant epidermal thickness [36,37]. This substantial expression of TGs may be a compensatory mechanism in response to proliferative signalling [36].

#### 3.1.2. Disruption of Intercellular Connections

Skin barrier dysfunction in psoriasis also stems from the disruption of epidermal tight, gap and adherens junction proteins. These proteins connect adjacent cells, differentiate the apical and basal aspects of the cell membrane, and regulate paracellular molecular passage. In the epidermis, TJ proteins are typically expressed exclusively in the stratum granulosum and upper stratum spinosum. However, in psoriatic lesions, the TJ proteins ZO-1, occludin and claudin-4 are highly expressed outside of the stratum granulosum [38,39]. TJ proteins also have reduced expression throughout skin lesions, which points to the global disturbance of TJ function [33,40]. This disturbance likely contributes to the heightened transepidermal water loss and reduced hydration found in psoriatic lesions [41]. The induction of keratinocytes with IL-17 or IL-1β down-regulates keratinocyte adhesion proteins, so inflammatory signalling may be the cause of dysregulated TJ function [42,43]. Furthermore, TJ dysregulation is found in the early stages of psoriatic lesion development, which points to the possibility that barrier dysregulation contributes to the onset of psoriasis [39].

E-cadherin is a crucial part of adherens junctions. Immunohistochemical studies have revealed that the expression of E-cadherin is reduced in epidermal basal and the upper granular layers of skin lesions [44]. Furthermore, epidermal E-cadherin is also damaged in psoriatic lesions, which feature an increased presence of truncated e-cadherin, and a decreased presence of the functional form of the protein [44]. Corneodesmosin, a protein that facilitates corneocyte adhesion, is, additionally, dysregulated in skin lesions of psoriasis. It is observed deeper than in healthy skin, with expression beginning in the stratum spinosum instead of the upper stratum granulosum [45]. Further, corneodesmosin in the stratum corneum of healthy skin is typically degraded, but it is intact in the lesional skin of psoriasis [46].

Connexins are the structural building blocks of gap junctions. Connexin 26 (CX26), one of the smallest connexins, is almost entirely absent in healthy epidermis but is one of the most highly upregulated proteins in psoriatic plaques [47]. *GJB2*, which encodes CX26, is the 98th most upregulated gene detected, and its overexpression is used as a marker of genetic predisposition in psoriasis [48,49]. The inhibition of CX26 expression diminishes inflammatory pathways and may be therapeutically useful in psoriasis [50].

#### 3.1.3. Dysregulation of the Lipid-Rich ECM of the Stratum Corneum

Barrier disturbance is not only from dysfunction in keratinocyte adhesion, but also from the dysregulation of the stratum corneum ECM. The most common lipid in the stratum corneum, ceramide, has multiple subtypes. The prevalence of ceramide subtypes differ in psoriatic lesions, in a manner which is correlated with transepidermal water loss [51]. The total amount of ceramide in keratinocytes and fibroblasts is not reduced in psoriatic lesions, suggesting that this effect is from the dysregulation of the ceramide subtype, rather than a reduction of the lipid [52]. Ceramide dysregulation may also potentiate psoriatic inflammation, as mice with aberrant ceramide metabolism spontaneously develop IL-17-mediated inflammation and a psoriatic phenotype [53].

Other lipids are also dysregulated in the stratum corneum of those with psoriasis. Skin lesions have reduced the levels of short chain fatty acids and increased the levels of cholesterol, compared to healthy skin [54]. Cholesterol accumulates in keratinocytes following IL-17 signalling, so its dysregulation is likely secondary to the chronic psoriatic inflammatory response [55]. Interestingly, cholesterol accumulation in keratinocytes also facilitates the inflammatory response by potentiating the expression of IL-17 induced genes [55]. Overall, psoriatic lesions have elevated lipid levels [54]. These findings differ from the stratum corneum lipid dysregulation in atopic dermatitis lesions, which feature reduced levels of total skin lipid and ceramide [56]. However, the relative proportions of lipids in uninvolved skin in those with psoriasis, atopic dermatitis and no skin disease are comparable [57].

#### 3.1.4. Other Contributing Factors

Aquaporin-3 (AQP3) is an epidermal water/glycogen channel protein that facilitates skin hydration [58]. Several studies have found AQP3 expression is down-regulated in psoriatic lesions and is abnormally localised to the keratinocyte cytoplasm, which may contribute to skin barrier disruption and dehydration in psoriasis [59,60,61].

The role of fibroblasts in the dermis is mainly to produce the fibrous ECM. Their signalling also promotes keratinocyte proliferation and differentiation and is dysregulated in psoriasis [62]. Fibroblasts from psoriatic lesions release reactive oxygen species that are internalised by keratinocytes in vitro and induce proliferation [63]. Further, fibroblasts treated with psoriatic cytokines in vitro produce epiregulin, a ligand for the epidermal growth factor receptor that compels keratinocyte proliferation [64]. Fibroblasts in skin lesions also down-regulate proteins involved in cellular adhesion, providing another means by which fibroblasts may contribute to physical barrier disruption [65].

The physical skin barrier is, therefore, a complex system enacted by the epidermis, which acts to protect the body from environmental insults. Psoriatic states compel both structural and functional changes that drive the development of skin lesions.

### 3.2. Immune Barrier Dysregulation

**Figure 2:**
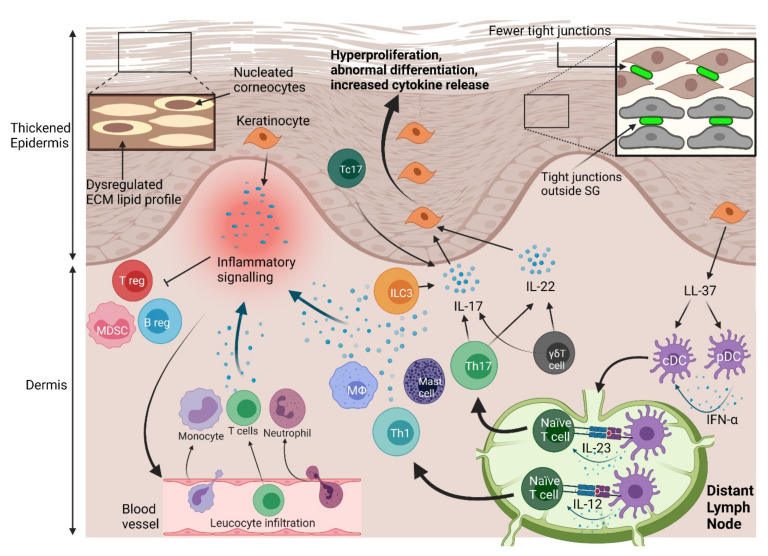
Physical and immune barrier disruption in psoriasis. Hyperproliferation of keratinocytes contributes to a thicker epidermis. This process is highlighted by increased expression of proliferation markers. Physical barrier function is compromised by decreased tight junction protein expression, and expression of tight junctions outside of their typical place in the stratum granulosum. Ceramide production is dysregulated in the lipidrich ECM of the stratum corneum, which also adds to physical barrier disruption. Aberrant epidermal differentiation is marked by the abnormal presence of nuclei in some corneocytes. Keratinocytes proliferate strongly in response to inflammatory cytokines, including interleukin (IL)-17 and IL-22. IL-17 is mostly produced by type 3 innate lymphoid cells (ILC3s), T helper 17 (Th17) cells, γδ T cells and the cytotoxic T cell (Tc) subtype Tc17 cells. IL-22 is produced largely by Th17 and γδ T cells. Th17 cells differentiate in response to IL-23 signals from dendritic cells (DCs), which can also compel the differentiation of Th1 cells by releasing IL-12. Plasmacytoid (p)DCs and conventional (c)DCs are activated by cathelicidin (LL-37) produced by keratinocytes. Cytokines released by keratinocytes, the aforementioned immune cells, neutrophils, macrophages (Mφ), and mast cells create an inflammatory environment that compels infiltration of T cells, neutrophils, and monocytes from blood vessels. Inflammatory mediators also inhibit regulatory T (Treg), regulatory B (Breg) cells, and myeloid derived suppressor cells (MDSCs). These dysregulated physical and immune responses lead to disrupted skin barrier function and, finally, psoriasis.

Psoriasis is primarily mediated by the dysregulation of the immune skin barrier (Figure 2). Innate immune cells respond to inflammatory triggers by releasing proinflammatory cytokines and activating adaptive immune responses. After activation, the adaptive immune system generates a substantial inflammatory response, also largely mediated by cytokine release, which drives keratinocyte hyperproliferation. In response, keratinocytes produce autoantigens and cytokines, further promoting inflammation. Immune responses are potentiated by autoantigens, but it remains unclear whether they trigger psoriatic inflammation, or whether autoimmunity is secondary to other inflammatory mechanisms. Additionally, inhibitory immune pathways in psoriasis are impeded, which enables the inflammatory response to be maintained, and develop into a chronic state.

#### 3.2.1. Keratinocytes Promote Inflammation

Keratinocytes trigger psoriatic inflammation by producing autoantigens—including cathelicidin (LL-37), a disintegrin and metalloprotease domain containing thrombospondin type 1 motif-like 5, the neolipids associated with phospholipase A2 group IVD (PLA2G4D), and Keratin 17 [66]. Keratinocytes, additionally, facilitate DC autoantigen recognition by producing polyamines which prevent degradation of autoantigenic RNA [67]. A list of all known and potential autoantigens is found in Table 1.

Keratinocytes further promote psoriatic inflammation by producing proinflammatory cytokines, including IL-6, IL-8, IL-25, IL-36, tumour necrosis factor (TNF)-α, C-X-C motif chemokine ligand 10 and chemokine ligand 2 (CCL2) [84,85]. Keratinocytes in psoriasis also substantially produce IL-1β, which activates γδT cells and also acts in an autocrine fashion, compelling other keratinocytes to produce chemokines that induce the immune cell infiltration of the dermis [86]. Keratinocytes treated with a psoriatic cytokine profile, additionally, produce proinflammatory exosomes that are endocytosed by neutrophils and induce them to produce neutrophil extracellular traps (NETs), IL-6, IL-8 and TNF-α. Mice with keratinocytes that cannot release these exosomes have less of a psoriatic phenotype than controls, highlighting the importance of this process in vivo [87].

Keratinocytes may also potentiate psoriatic inflammation by direct cell–cell contact with T cells. Keratinocytes treated with interferon (IFN)-γ can induce the differentiation of naïve T cells into T helper (Th) 1 and Th17 subtypes, in the absence of professional antigen presenting cells [88]. This effect is reliant on cell–cell contact mediated by interactions between CD58/CD2 and between intercellular adhesion molecule 1 and lymphocyte function associated antigen 1. Although T cells typically exist in separate compartments to epidermal keratinocytes, it is possible that they infiltrate the epidermis given the fact that the physical skin barrier is dysregulated in psoriasis.

#### 3.2.2. Inflammatory Immune Cells 

##### Altered Innate Immune Cell Function

Innate immune cells initiate psoriatic inflammation by producing cytokines like IL-17 and IL-22 that directly trigger keratinocyte proliferation and create a powerful proinflammatory milieu. We will detail the pathogenic roles of these cells in psoriasis (including dendritic cells, neutrophils, macrophages, and mast cells), highlighting recent findings, and suggesting explanations for contradictory evidence in the field. We will also discuss innate immune cells with critical roles in psoriasis that are still being characterised (including LCs, ILCs and γδ T cells).

Dendritic cells are often considered the most important innate immune activator in the pathogenesis of psoriasis. The initial activation of the inflammatory milieu is likely mediated by pDCs, as they are found abundantly in early psoriatic skin lesions but are absent in chronic ones [89]. Furthermore, pDCs recognise self nucleic acids stabilised by antimicrobial peptides, and subsequently produce IFN-α [6]. IFN-α induces the maturation of cDCs, sensitises keratinocytes to IL-22 by compelling the upregulation of IL-22R and compels the conversion of naïve CD4+ cells into Th1 and Th17 cells [6,90]. Contrastingly, cDCs are associated with antigen presentation and are found in lymph nodes and colocalised with T cells in the dermis, so they are likely involved in autoantigen presentation in psoriasis [91,92]. In addition, cDCs can also release a myriad of cytokines, including TNF-α, IL-6, IL-12, IL-20, and IL-23 [93].

Langerhans cells are a DC subtype that are critical in the skin barrier, but their role in psoriasis remains unclear. There is no consensus on whether LCs are more or less prevalent in the epidermis of psoriatic lesions [6]. Two mouse models with depleted LCs have shown greater neutrophil infiltration in the late stages of pathology and more psoriatic symptoms, pointing to the conclusion that LCs are anti-inflammatory in psoriasis [94,95]. However, other studies in mice with an imiquimod induced psoriasis phenotype concluded that LC function is necessary for psoriatic inflammation [96,97]. This tension may be accounted for by the presence of LC subtypes—for instance, Singh et al. found that skin resident LCs were dispensable for psoriatic inflammation in mice with IL-23 induced disease, but myeloid-derived inflammatory associated LCs contributed to the inflammation [98]. More analysis of LC subtypes is needed to clarify their role in psoriasis. 

Neutrophils potentiate early psoriatic inflammation by releasing IL-1β, a cytokine that is predominant in early skin lesions [99]. Neutrophils, additionally, facilitate ongoing psoriatic inflammation by releasing proteases which activate the precursors of TNF-α and IL-36 [100]. They also release IL-17, but it is debated whether neutrophils produce IL-17 themselves, or whether they endocytose and store IL-17 produced by other cells and release it after inflammatory signalling [93]. The role of NETs in psoriasis has recently drawn focus in the field because their prevalence correlates with psoriatic disease activity and they compel inflammation in multiple other immune mediated diseases [100,101]. NETs can induce Th17 differentiation [102] and act as a reservoir for LL-37–nucleic acid complexes that activate several innate immune cells, including neutrophils themselves [103].

Macrophages primarily induce psoriatic inflammation by producing the cytokines TNF-α, IL-1β, IL-12 and IL-23 [86,104,105,106,107]. In mice with IL-23 induced psoriatic phenotypes, macrophages infiltrate into psoriatic lesions later than other leucocytes, suggesting they may have particular roles in maintaining psoriatic inflammation [104]. There is debate over whether macrophages in psoriatic lesions directly stimulate keratinocyte proliferation by producing IL-17. Macrophages stimulated by IL-23 in vitro produce IL-17 [108], however, macrophages in mice with IL-23 induced disease do not substantially release IL-17 [104]. Studies of ex vivo macrophages from human psoriatic lesions will clarify this tension.

Mast cells help to initiate psoriatic responses by inducing the early recruitment of pDCs into skin lesions [89] and by producing PLA2G4D—an enzyme which generates lipid autoantigens [76]. Mast cells also propagate psoriasis by producing IL-17, and being a primary producer of IL-22 in psoriatic lesions [109,110]. Despite these key roles in the pathology of psoriasis, there is no significant correlation between mast cell accumulation and pruritis (a major symptom of psoriasis), which limits the possibility of the success of a mast cell based treatment [111].

Innate lymphoid cells are a recently studied class of immune cells, and their role in mediating psoriasis pathology is still being explored. Their transplantation into mice with human grafted skin contributes to the formation of psoriaticlike dermatitis [112]. ILCs may be more important in IL-17 production than T cells because deleting T cells from two mouse models of psoriasis does not abolish skin hyperplasia, but deleting ILCs does [113]. The ILC3 subclass are disproportionately abundant in the blood and skin lesions of psoriasis patients and are classified by their production of IL-17 and IL-22, both cytokines that are key in the induction of keratinocyte proliferation [114,115]. Further investigation into means of targeting ILC3s may lead to novel psoriatic therapeutics.

γδ T cells are a subset of immune cells with innate and adaptive features. They have a T cell receptor (TCR) (using γ and δ subunits instead of the classical α and β subunits) but do not require TCR activation to proliferate or facilitate inflammation [116]. γδ T cells are key producers of IL-17 in psoriatic lesions, but can also produce IL-22 and TNF-α [107,117]. There is a large variety of γδ T cell subtypes with different prevalences in psoriatic and healthy individuals [118]. A future direction of the field is, therefore, to characterise the role of the γδ T cell subtypes to better understand their contribution to psoriatic inflammation. For instance, in mice with an imiquimod induced psoriasis phenotype, inflammation induces IL-17-producing Vγ4+ γδ T cells that have memory functions, which may play a role in the propagation or reactivation of psoriasis [119,120]. However, it is difficult to assess the importance of γδ T cell subtypes because findings in mice are not necessarily generalizable. All T cells in the healthy mouse epidermis, and approximately 54% of T cells in the dermis, are γδ T cells [121], numbers which are much lower in human skin [122,123].

The innate immune system, therefore, initiates psoriatic inflammation through cytokine release and the activation of adaptive immune responses. There is substantial redundancy in the pathways—multiple cells produce similar cytokine profiles (notably TNF-α, IL-17 and IL-22), and many cytokines induce similar inflammatory effects. Analysing these common pathways is helpful, as they are often the most important in the development of disease. However, the redundancy makes it difficult to target innate immune cells for therapeutic purposes. Nevertheless, novel research is highlighting the unique contributions of particular cells to psoriasis pathogenesis, which may be targets for future therapies. 

##### Excessive Activation of Adaptive Immune Response

Adaptive immune cells in psoriasis are activated by aberrant innate immune cell signalling, and subsequently release inflammatory mediators which potentiate psoriatic inflammation. Th17 cells and specialised CD8+ cytotoxic T cells (Tc) are particularly important because they substantially produce IL-17, thereby compelling keratinocyte proliferation. Analysing the unique cell subtypes and signalling pathways of the adaptive immune system may reveal novel therapeutic targets, specific to inflammatory disease.

Th 17 cells are one of three well characterised Th cell subtypes [124]. Th cells can be classified by the major cytokines they produce—Th1 cells (IFN-γ), Th2 (IL-4) and Th17 (IL-17) [125]. Initially, Th1 cells were considered the crucial subtype in promoting psoriatic lesions, particularly in their production of the proinflammatory cytokines TNF-α, IL-2, and IFN-γ [126]. The importance of Th1 functions is also emphasised by the fact that TNF-α inhibitors and methotrexate (both psoriasis treatments) reduce the expression of Th1 cytokines and reduce Th1 prevalence in those with psoriasis [127,128,129].

It is now thought that Th17 cells are the critical adaptive responders in psoriatic lesions, particularly due to their substantial production of IL-17. IL-17 is a crucial cytokine in inducing keratinocyte proliferation and compelling keratinocyte production of antimicrobial peptides, chemokines, and members of the IL-1 cytokine family [130,131]. Further, Th17 cells can produce the proinflammatory cytokines IL-22, IL-26 and IFN-γ, and the chemokine CCL20 [132]. CCL20 compels the infiltration of Th17 cells, which then produce more CCL20, ultimately creating an inflammatory positive feedback loop that drives psoriasis pathology [133]. Th17 cells remain active in lesions even after the resolution of symptoms post-treatment, so they may have a role in disease recurrence [134]. The importance of Th17 cells is highlighted by the success of anti-IL-23 psoriasis therapies, a cytokine that induces the differentiation of Th17 cells [135].

Th17 cells also have noninflammatory, homeostatic functions, and so are separated into pathogenic and beneficial subtypes [136]. Unsurprisingly, pathogenic type Th17 cells are the subtype enriched in psoriatic lesions and peripheral blood [137]. Therapies specifically targeting pathogenic Th17 cells may, therefore, remove a promoter of psoriatic inflammation without removing homeostatic Th17 responses. The CRISPR mediated targeting of pathogenic Th17 cells has already been successful in mice [138], and, so, is a promising direction in the field. 

CD8+ T cells in psoriatic lesions can release multiple proinflammatory cytokines, including IL-17, IL-21, IL-22, TNF-α and IFN-γ [139,140]. The traditional cytotoxic role of CD8+ T cells may also be associated with psoriasis pathology, as CD8+ T cells that produce the cytotoxic mediator granulysin outnumber CD8+ T cells that do not in the blood and skin of psoriasis patients [141]. There are 11 subtypes of CD8+ T cells in the skin when analysed by RNA profile [142]. The two subclasses most uniquely found in psoriatic lesions produce IL-17, named Tc17 cells [142]. The prevalence of Tc17 cells in psoriatic lesions correlates with disease duration, further evidencing their importance [143]. Tc17 cells from lesions are also more active ex vivo than their counterparts—they proliferate more with Treg inhibition and have increased cytotoxic capacity [139]. Moreover, the memory subtypes of Tc17 cells are enriched in the epidermis of psoriatic lesions, so their IL-17 production likely directly stimulates local keratinocytes to proliferate [144]. Put together, this evidence suggests Tc17 cells contribute to psoriatic disease, and so could be a useful, specific target in future treatments.

#### 3.2.3. Immunosuppressing Cells

The pathology of psoriasis is associated with the activation of proinflammatory responses and also with the inactivation of anti-inflammatory responses. Regulatory T (Treg) cells, regulatory B (Breg) cells and myeloid derived suppressor cells (MDSCs), in particular, fail to enact their typical homeostatic roles in psoriasis.

Treg cells are the best characterised immunosuppressing cell. The deletion of Treg cells in mice with imiquimod induced psoriasis phenotypes leads to increased infiltration of γδ, CD4+ and CD8+ T cells, the increased presence of IL-17 and TNF-α, as well as increased severity of skin lesions [145,146]. Further, several treatments of psoriasis operate by stimulating the proliferation and action of Treg cells, highlighting their importance in pathogenesis [147,148]. These findings raise the question: if Treg cells can prevent psoriatic inflammation, why do they not prevent psoriasis in those who have the disease? Treg cells are reported to have increased prevalence in psoriatic skin by most studies, so their dysfunction is unlikely due to insufficient prevalence [149]. It is more likely that Treg cells in skin lesions have impeded suppressive function.

Psoriatic Treg cells have limited capacity to inhibit CD4+ T cell proliferation, proliferate less in response to CD3/CD28 TCR stimulation, and express higher levels of TNF-α and IFN-γ ex vivo [150,151]. Tregs are likely suppressed in psoriasis because of the pro-inflammatory cytokine milieu, particularly IL-6 [152] and IL-21 [153], which operate through the adaptor molecule signal transducer and activator of transcription 3 (STAT3). Inhibiting STAT3 partially restores the suppressive function of psoriatic Treg cells [154]. Treg dysfunction in psoriasis is relatively uncharacterised compared to other immune cells, so more analysis into disrupted regulatory signalling mechanisms is needed.

Breg cells are the key B cell subtype studied in the pathogenesis of psoriasis. These cells inhibit immune responses particularly by producing the anti-inflammatory cytokine IL-10 [155]. They have a reduced prevalence in the circulation of psoriasis patients, and their progenitor cell has an increased prevalence, suggesting psoriatic states could prevent their differentiation [156]. In two mouse models, Breg presence is associated with a reduction in skin lesion severity, less production of IFN-γ and IL-17, decreased Th17 differentiation, and increased Treg differentiation [157,158]. These findings suggest psoriasis pathogenesis may involve Breg cell dysregulation, and that future therapies may involve their reconstitution.

Myeloid derived suppressor cells are more prevalent in skin lesions and circulation in those with psoriasis compared to controls [159]. Psoriatic MDSCs inhibit T cell proliferation and cytokine secretion ex vivo, pointing to a role in inhibiting inflammation [160,161], however, this ability is reduced when compared to normal MDSCs [161]. Mice with imiquimod induced psoriasis phenotype show the conflicting roles of MDSCs. Kim et al. found that the administration of MDSCs into mice with imiquimod induced disease reduced skin lesion severity and proinflammatory cytokine load [162]. However, Chen et al. found that depleting mouse MDSCs in the same model suppressed psoriatic lesion thickness and severity, implying MDSCs induce psoriasis [159]. These opposing actions of MDSCs might be explained by the existence of multiple MDSC subtypes with different roles and heterogeneous suppressive mechanisms among psoriatic patients [161]. More analysis is needed to investigate MDSC classes and, importantly, why they fail to inhibit psoriatic inflammation.

Psoriasis features a complex set of interactions between keratinocytes and immune cells, which each play a role in potentiating inflammation. Particularly, the activation of inflammatory immune responses and the inhibition of regulatory adaptive immune responses are crucial. The analysis of innate and adaptive immune cell subtypes has identified unique pathways that potentiate psoriatic inflammation, and also provide specific targets for potential future psoriasis therapy. The current understanding of psoriasis pathology is mostly from ex vivo studies using skin lesions, in vitro studies using keratinocytes and in vivo studies using mouse models with psoriasislike inflammation. Mouse models are useful as they replicate skin lesion histology and mimic psoriatic inflammation [163], but are imperfect as mouse skin is thinner and has different structural layers, base stress levels and mechanical properties when compared to human skin [163,164]. An awareness of these dissimilarities is critical to correctly analyze and translate preclinical results to human applications.

## 4. Contributors to Barrier Dysregulation in Psoriasis

Psoriasis only develops in some susceptible individuals, and results from the interaction between genetic and environmental factors. Twin studies have established that approximately 2/3 of psoriasis susceptibility is genetic [165,166]. An extensive series of environmental contributors to psoriasis has also been described, but the pathological links to psoriasis of these contributors are often poorly defined. Here, we describe key genetic, epigenetic, and environmental risk factors for psoriasis.

### 4.1. Genetic Aberrations 

#### 4.1.1. Susceptible Genes

Genomewide association studies have revealed psoriasis occurs in people with particular susceptibility alleles. Over 80 genetic loci associated with the onset of psoriasis have been identified [167]. They largely map to keratinocyte and immune signalling pathways (Table 2). Human leukocyte antigen (HLA) genes are the most substantial contributors to psoriasis genetic susceptibility, particularly the HLA-C*06:02 allele [167]. HLA-C*06:02 presence in psoriasis patients is associated with greater plaque severity (measured by the extent of inflammation and body area covered) and earlier disease onset [168,169]. HLA-C corresponds to the major histocompatibility complex class I, and so is involved in antigen presentation to CD8+ T cells [74,170]. It is likely that HLA-C*06:02 alleles impart an atypical capacity to present autoantigen to CD8+ T cells, which could initiate psoriatic inflammation. [171,172].

Some psoriatic genetic susceptible loci regulate the physical skin barrier, which suggests barrier disruption may be a predisposing factor for psoriasis. Krüppel-like factor 4 (KLF4) is a transcription factor associated with psoriasis [173]. KLF4 is required to develop cornified envelopes that help to structure the lipidrich physical skin barrier [174]. The gene cluster that encodes the late cornified envelope proteins is also a susceptible locus in psoriasis [175].

**Table 2:** Key pathways to which psoriasis genetic susceptibility factors localize.

Biological Pathway	Representative Susceptibility Genes	Possible Role/s in Psoriasis	Target Drugs
HLA mediated antigen presentation	HLA-C*06:02, HLA-A, HLA-B and HLA-DQ [176,177].	Facilitates presentation of autoantigens [171,172].	No targeted drug currently. HLA-C*06:02+ individuals respond better to the anti-IL-12/IL-23 biologic ustekinumab [178].
NF-kB signalling	FASLG, IKBKE, NFKBIA, REL, SLC44A2, TNFAIP3, TNIP1, TRAF3IP2 [179] and CARD14 [180].	Elevates innate immune responses, activates T helper cells and reduces keratinocyte death [181].	Fumarate and apremilast inhibit NF-kB activation [182].
Th17 cell activation	IL23R, IL23A, IL12B [183].	Compels keratinocyte proliferation and promote psoriatic inflammation [132].	Biologics targeting IL-23 (tildrakizumab, guselkumab, risankizumab, and ustekinumab), novel RORγ inhibitors and JAK inhibitors [184,185].
Skin structure proteins	The LCE3 gene cluster, KLF4, COL6A5 and COL8A1 [173,175,186].	LCE3 and KLF4 genes facilitate cornified envelope production, and their variants contribute to barrier dysfunction [187]. COL6A5 regulates cell adhesion and proliferation and COL8A1 mediates vascularisation [186].	Topical calcitriol may operate by upregulating LCE genes [188].
Keratinocyte proliferation and differentiation	Keratins 6, 10, 14, 16 and 17 [26,189]. PDCD5, PTEN and CHUK [179,190].	Keratin 10, 14, 16 and 17 variants, reduced keratin 1 and 10 levels and elevated keratin 6, 16 and 17 levels associate with keratinocyte hyperproliferation and aberrant differentiation [23,26,189]. PDCD5 is hypermethylated, reducing its expression and capacity to facilitate apoptosis [190]. IKKa (the protein CHUK encodes) and PTEN typically regulate differentiation and proliferation, respectively [191,192].	Topical calcineurin inhibitors and vitamin D receptor agonists, such as calcitriol and retinoids, prevent atypical keratinocyte proliferation and differentiation [193].
Type 1 IFN signalling	DDX58, IFIH1 and RNF114 variants [180,194].	Sensitises keratinocytes to IL-22, induces the maturation of cDCs and facilitates the differentiation of naïve CD4+ cells [6,90].	UVB phototherapy downregulates IFN signalling pathways [195]. Novel IL-36 inhibitors may modulate IFN responses [196].

Abbreviations—CARD14: caspase recruitment domain family member 14; CCL: chemokine ligand; CHUK: component of inhibitor of nuclear factor kappa B kinase complex; DDX58: DExD/H-Box helicase 58; FASLG: fas ligand gamma; HLA: human leukocyte antigen; IFIH1: interferon induced with helicase C domain 1; IKK: IκB kinase; IKBKE: inhibitor of NF-kB kinase subunit epsilon; IL23R: interleukin-23 receptor; JAK: Janus kinase; KLF4: Krüppel-like factor 4; LCE: late cornified envelope; NF-kB: nuclear factor-κB; NFKBIA: NF-kB inhibitor alpha; PTEN: phosphatase and tensin homolog; PDCD5: programmed cell death 5; RNF114: ring finger protein 114; RORγ: retinoic acid receptor-related orphan receptor gamma; SLC44A2: solute carrier family 44 member 2; TNFAIP3: TNF alpha induced protein 3; TNIP1: TNFAIP3 interacting protein 1; TRAF3IP2: TRAF3 interacting protein 2; UVB: ultraviolet-B.

#### 4.1.2. Epigenetic Modifications 

Epigenetic modifications have also been found to be associated with psoriasis. For example, DNA methylation is centred in gene loci associated with psoriatic states, including caspase recruitment domain family member 14 and HLA-C [197,198]. DNA methylation profiles can be used to stratify psoriasis patients by clinical features, including age of disease onset and IL-22 copy number variation, allowing the potential future use of epigenomics in classifying psoriasis patients for diagnostic or treatment purposes [199]. Keratinocyte stem cell dysregulation and proliferation has also been associated with DNA hydroxymethylation [200]. Moreover, psoriatic epigenetics encompasses modifications other than DNA methylation—several histone modifications and noncoding RNA changes have been described [201]. However, it is difficult to establish whether epigenetic modifications in diseased states initiate pathology or are a result of pathology, so their importance is unclear. The discussed findings are a few of the studied epigenetic modifications, which are thoroughly reviewed here [201,202].

There are several key gaps in our knowledge concerning psoriasis genetic susceptibility, with meta-analyses on the genetic contribution to psoriasis failing to account for 70% of cases [179]. This may be related to the role of undiscovered and/or rare psoriasis-associated alleles. Further, there has been a lack of genotyping of psoriasis patients outside of Europe and East Asia [167]. This is particularly important as psoriatic susceptibility alleles are different between European and East Asian populations, so they will likely be different in other populations too [176].

### 4.2. Environmental Factors 

#### 4.2.1. Imbalances in the Gut/Skin Microbiome

Gut microbiome alterations at the phylum, family, genus, and species levels are strongly associated with psoriasis. Despite inconsistencies in some results, an index using microbiome data has been created that can predict whether patients have psoriasis. This index has a sensitivity of 0.78 and a specificity of 0.79 [203], although this requires further validation for use of diagnosis [203]. It will be challenging for microbiome profiles to exceed the accuracy of current clinical psoriasis diagnosis. However, similar approaches may be useful in identifying susceptible individuals before they develop skin lesions, and flag them for early treatment.

Microbiota produce short and medium chain fatty acids which can regulate immune responses, which may explain their link to psoriasis pathogenesis [204]. For example, alterations in the gut microbiome correlate with increases in inflammatory markers, including the IL-2 receptor and the complement 3 protein [205]. Additionally, the gut microbiome from psoriasis patients has a lower gene expression of proteins which synthesise butyrate (an anti-inflammatory short-chain fatty acid) than healthy controls [206,207]. 

The prevalence of some operational taxonomical units in the skin microbiome of psoriatic lesions is also significantly altered compared to healthy skin [208,209,210]. The skin microbiota can regulate immune responses by being directly immunogenic or by preventing pathogenic growth [211]. *S. aureus* disproportionately colonises psoriatic lesions [212], and is particularly notable as its superantigens can induce IL-17 and IFN-γ responses [213].

Despite these findings, studies analysing the prevalence of specific taxonomical units in the gut and skin microbiome yield conflicting results [214]. There are also contradictory findings concerning whether the gut microbiome is more or less diverse in those with psoriasis, with more studies finding a reduction in biodiversity [215]. These variations could be explained by a number of confounding factors, including variation in subjects’ disease activity, analyses of different gene regions, and standardising for obesity [216]. Furthermore, relatively few studies incorporating longitudinal cohorts have been conducted, reducing the power of conclusions made.

#### 4.2.2. Infections

Infections also initiate and exacerbate psoriatic lesions. *Streptococcal* pharyngeal infections are strongly associated with guttate psoriasis but can also exacerbate chronic plaque psoriasis to such an extent that tonsillectomy is a viable management option for some treatment resistant psoriasis [169,217,218]. Interestingly, *streptococcal* infections are more common in those with the psoriasis susceptibility HLA-C*06:02 allele [219], which may explain why psoriasis patients have an increased burden of *streptococcal* infections than healthy controls [169]. Individuals with psoriasis are also disproportionately likely to develop *H. pylori* and oral *C. albicans* infections [220,221]. However, these findings may be a result of confounders or from psoriasis patients being more susceptible to infection. *H. pylori* may potentiate psoriasis by inducing CD4+ T cell responses [222], while *C. Albicans* elicits antibody and Th17 responses [223]. Patients with treated *H. pylori* also have a lower severity of psoriasis than untreated individuals, further highlighting their association [222].

#### 4.2.3. Skin Damage

Skin injury in susceptible individuals often initiates psoriatic lesions. This finding, known as the Koebner phenomenon, is recapitulated in other skin diseases [224] and may be mediated by injury inducing the production of type 1 IFNs, TNF-α, IL-6 and IL-36 in keratinocytes [225,226]. Moreover, mechanical stretch also contributes to psoriatic inflammation [227]. Keratinocytes in vitro and in two mouse models exposed to stretching produce more markers of cell proliferation, reduced markers of differentiation and more soluble inflammatory mediators. This explains why psoriasis disproportionately forms in areas of high tension, notably on extensor surfaces such as the elbow and knee.

Itch (or pruritis) is a common symptom of psoriasis, affecting between 62 and 97 percent of patients [228]. The pathophysiology of itch in psoriasis is complex and not fully elucidated, but includes contributions from neuropeptides, neurotransmitters, cytokines, hormones, and the vascular system [229]. Itching also exacerbates skin barrier dysregulation in psoriatic lesions by compelling epidermal inflammation, exemplifying the Koebner phenomenon [228]. Interestingly, pruritis is common in nonlesional skin of those with psoriasis, and so can initiate the onset of psoriatic plaques [230]. Scratching also activates sensory neurons and compels their release of proinflammatory neuropeptides, which may aggravate skin barrier dysregulation in psoriasis [231].

In some cases, medications may exacerbate or initiate skin lesions, notably β-blockers, antimalarials, lithium, nonsteroidal anti-inflammatories and tetracycline [232]. Rarely, vaccinations can also contribute to psoriasis flares [233,234].

Psoriasis is a complex disease with multiple exacerbating factors, which combine to trigger skin inflammation in those with genetic predispositions. More analysis of the biochemical pathways underlying the predisposing factors discussed will uncover targets for therapeutic options and biomarkers to facilitate diagnosis.

## 5. Psoriasis Current Treatment and Future Directions

A major goal of psoriasis treatment is to correct the dysregulation of skin barrier structure and function in lesions. The current understanding of physical and immune barrier aberrations in psoriasis has contributed to the advent of treatment options that can effectively manage many individuals with the disease [11,184]. Broadly, there are five classes of treatment—topical therapy, phototherapy, non-biologic systemic therapy, small molecule inhibitors and biologics. The introduction of biologics has been particularly important in improving outcomes and quality of life in those with severe psoriasis. We will briefly detail the mechanisms behind current treatment options, and then look to future directions of psoriasis therapy, which include finding novel targets and creating novel treatment approaches to maximise the effectiveness of current drugs.

### 5.1. Current Treatments 

Topical treatments, including corticosteroids, vitamin D analogues, calcineurin inhibitors and keratolytics, are less effective than other therapies but are convenient and have few adverse effects, so are typically used to treat mild psoriasis [11]. Corticosteroids largely act on the immune skin barrier and keratolytics act on the physical skin barrier. Vitamin D analogues and calcineurin inhibitors modify both the physical and immune barriers of skin. They both enhance differentiation and limit proliferation in keratinocytes [193,235]. Calcineurin inhibitors also supress the proliferation of T cells, whereas vitamin D analogues inhibit the production of cytokines in macrophages and DCs [193,235].

Phototherapy is used in mild or treatment resistant psoriasis and similarly modulates the skin’s physical and immune barriers [11]. This therapy induces keratinocyte and T cell apoptosis, biases naïve CD4+ T cells away from Th1 or Th17 responses and compels the proliferation of Treg cells [236].

Nonbiologic systemic therapeutics (including methotrexate, cyclosporine and acitretin) and small molecule inhibitors (including apremilast) are used for more substantial psoriasis [11,237]. Multiple small molecule inhibitors of Janus kinase (JAK) inhibitors are also being developed, which block the intracellular signalling downstream of IL-23 and other key psoriatic cytokines [238]. However, biologic therapies are more effective treatments for moderate and severe psoriasis than nonbiologic or small molecule agents—including the most characterised JAK inhibitor tofacitinib [237].

Currently approved biologics target IL-17, IL-23 and TNF-α—all key cytokines in the pathogenesis of psoriasis [184]. The unique biologic ustekinumab also targets IL-12, as it binds a shared subunit of IL-12 and IL-23 [11]. The effectiveness of psoriasis drugs is often reported by the relative risk of achieving a 90% reduction in psoriasis lesion severity in moderate–severe psoriasis patients, compared to controls. For biologics, the relative risk ranges from 14-31, whereas this number is 3.3 in small molecule inhibitors, and 6.5 in non-biologic systemic treatments [237]. Patients on biologics also report the highest treatment satisfaction rates of the drug options [239]. Anti-IL-17 agents as a class outperform other biologic classes, but there is no consensus on which specific drug is superior, or which has fewer side effects [237].

Additionally, the use of emollients as an adjuvant treatment can benefit all individuals with psoriasis [240]. Emollients restore physical barrier function by forming a lipid-rich layer that prevents water loss from the skin, and by facilitating water binding in the stratum corneum [241]. Their application to psoriatic plaques reduces transepidermal water loss, plaque scaling and pruritis, and also normalises epidermal markers of differentiation and proliferation [242,243]. Emollients are particularly used in conjunction with topical steroid therapy, as they increase drug penetration [242].

### 5.2. Future Directions of Treatment 

Despite the advent of new treatments, the majority of psoriasis patients are consistently found to be dissatisfied with their management [239]. This is due to ineffectiveness (particularly in those not on biologic therapy) and inconvenience (particularly in those on biologic therapy). There is, therefore, scope to develop more convenient and effective treatments, which may be achieved by finding new therapeutic targets or by using novel approaches to improve current treatments.

Due to the relative improvement of biologic therapy, a natural next step in developing new therapies for psoriasis is to target novel inflammatory mediators that function in multiple psoriatic pathways.

Several therapeutics are being developed to target IL-36, a cytokine that impedes the differentiation of keratinocytes and amplifies the effect of IL-17 on keratinocytes [244,245,246]. The most advanced is spesolimab, which induced an 80% improvement in lesion severity with no serious side effects in a phase I trial in patients with generalised pustular psoriasis, with a phase II trial being planned [247,248]. However, it remains unclear if targeting IL-36 is effective in chronic plaque psoriasis, particularly considering it failed to show significant improvement in patients with pustular psoriasis in an initial phase II trial [249]. Other trials have been conducted into biologics that target most of the cytokines previously described in the pathogenesis of psoriasis—including IL-1, IL-6, IL-12, IL-20, IL-22 and IFN-γ— and none substantially reduced psoriatic inflammation [250,251,252].

Novel biologics could also target chemokines and their receptors. Targeting chemokines has been effective in reducing psoriatic inflammation in several mouse models [253], and an anti-CCL20 antibody was effective at reducing chemokine receptor 6 positive cell infiltration into skin blisters in a human trial [254]. However, this antibody induced severe vascular and organ inflammation when used over 26 weeks in a monkey model, so novel antichemokine biologics may be needed [255].

Another potential target in psoriasis therapeutics is angiogenesis. Angiogenesis is an important step in the disruption of the epidermal barrier as it mediates oxygen access for proliferating keratinocytes and immune cell infiltration [256]. Inhibition of vascular endothelial growth factor (VEGF) A, a major regulator of physiological and pathological angiogenesis, reduces psoriatic inflammation in several mouse models and case reports [257]. However, there have also been multiple reports of the small molecule inhibitors of the VEGF receptor exacerbating psoriasis [258,259,260], so clinical trials are needed to assess the viability of this therapeutic strategy.

Other than creating new therapeutics, a different means of treating psoriasis is to increase the efficacy of currently developed therapeutics. One key limitation of biologics at present is their cost, which limits availability and their approval for subsidisation. However, novel modified antibodies are being generated which could be cheaper to produce and have improved efficacy [261]. One example is the nanobody sonelokimab, a trivalent molecule that binds IL-17A, IL-17F and albumin. It is composed of a single antibody variable domain, which makes it theoretically easier to produce, less degradable and better at skin penetration. Initial studies have shown its effectiveness in treating psoriasis [262].

Additionally, since current therapeutics are useful in some patients but not others, a means of knowing which drug will likely help which patient would improve their effectiveness. Such personalised medicine approaches have mostly stratified patients by genetic polymorphisms [263], particularly by the HLA-C*06:02 allele. For example, patients with the HLA-C*06:02 allele respond better to ustekinumab [178] and patients without HLA-C*06:02 respond better to the anti-TNF-α biologic adalimumab [264]. Novel approaches will involve separating by nongenetic biomarkers such as IL-19 levels, which correlates with disease severity and treatment response, so may be able to stratify patients and guide their treatment approaches [265]. Analyzing lesion and blood samples with machine learning or multiomics techniques are also a promising means of predicting response before and shortly after treatment initiation, in order to optimise choice of therapy [266].

The treatment of psoriasis has been relatively successful, as characterisation of disease pathology has allowed for the development of effective therapeutics for all spectra of psoriasis. However, there is scope to improve the convenience, cost, and efficacy of treatments. Further characterisation of barrier dysfunction in psoriasis will help to develop more effective therapeutic targets and approaches.

## Figures and Tables

**Table 1:** Autoantigens found in the pathogenesis of psoriasis.

Autoantigen	Traditional Function	Autoantigenic Function in Psoriasis
Cathelicidin (LL-37)	Antimicrobial peptide that induces innate immune cell response [68].	Activates DCs to release IFN-α [69,70]. Stimulates keratinocytes to produce IFN-α and IFN-β [71]. Activates CD4+ and CD8+ T cells [72].
ADAMTSL5	Regulates extracellular matrix microfibrils [73].	Activates CD8+ T cells, compelling IFN-γ and IL-17 production [74].
Lipids produced by PLA2G4D	PLA2G4D metabolizes lipids, producing linoleic acid [75].	Stimulate T cells to produce IL-17 and IL-22 [76].
Keratin-17	Plays roles in wound healing and tissue development [77].	Induces CD8+ T cells from psoriasis patients to release IFN-γ [78].
Ezrin, Maspin, HSP27, PRDX2 (keratinocyte produced proteins)	Modulate cytoskeleton regulation, inhibit proteases, promote chaperoning and enhance antioxidation, respectively [79].	Autoantibodies against these proteins have been identified. Maspin and PRDX2 induce psoriatic T cell IFN-γ release. Autoantigenic function may stem from sequence homology with streptococcal peptides [79].
hnRNP-A1	Regulates mRNA transcription and processing [80].	Provokes an autoantibody response [81]. Facilitates immunogenic RNA–amine complex entry into DCs [67].
Lysozyme, β-Defensins 2/3	Antimicrobial agents [82].	Bind self-DNA and activate pDCs to produce IFN-α [83].

Abbreviations— ADAMTSL5: a disintegrin and metalloprotease domain containing thrombospondin type 1 motif-like 5; cathelicidin: LL-37; DC: dendritic cell; hnRNP: heterogeneous nuclear ribonucleoprotein; HSP: heat shock protein; IFN: interferon; IL: interleukin; PLA2G4D: phospholipase A2 (PLA2) group IVD; PRDX2: peroxiredoxin-2.

## References

[1] Wong R., Geyer S., Weninger W., Guimberteau J.-C., Wong J.K. (2016). The Dynamic Anatomy and Patterning of Skin. Exp. Dermatol..

[2] Madison K.C. (2003). Barrier Function of the Skin: “La Raison d’Être” of the Epidermis. J. Investig. Dermatol..

[3] Bäsler K., Bergmann S., Heisig M., Naegel A., Zorn-Kruppa M., Brandner J.M. (2016). The Role of Tight Junctions in Skin Barrier Function and Dermal Absorption. J. Control. Release.

[4] Bäsler K., Brandner J.M. (2017). Tight Junctions in Skin Inflammation. Pflügers Archiv..

[5] West H.C., Bennett C.L. (2018). Redefining the Role of Langerhans Cells as Immune Regulators within the Skin. Front. Immunol..

[6] Wang A., Bai Y. (2020). Dendritic Cells: The driver of Psoriasis. J. Dermatol..

[7] Chambers E.S., Vukmanovic-Stejic M. (2020). Skin Barrier Immunity and Ageing. Immunology.

[8] Kabashima K., Honda T., Ginhoux F., Egawa G. (2019). The Immunological Anatomy of the skin. Nat. Rev. Immunol..

[9] Ali N., Rosenblum M.D. (2017). Regulatory T Cells in Skin. Immunology.

[10] Greb J.E., Goldminz A.M., Elder J.T., Lebwohl M.G., Gladman D.D., Wu J.J., Mehta N.N., Finlay A.Y., Gottlieb A.B. (2016). Psoriasis. Nat. Rev. Dis. Primers.

[11] Armstrong A.W., Read C. (2020). Pathophysiology, Clinical Presentation, and Treatment of Psoriasis: A Review. JAMA.

[12] Obradors M., Blanch C., Comellas M., Figueras M., Lizan L. (2016). Health-Related Quality of Life in Patients with Psoriasis: A Systematic Review of the European Literature. Qual. Life Res..

[13] Takeshita J., Grewal S., Langan S.M., Mehta N.N., Ogdie A., Van Voorhees A.S., Gelfand J.M. (2017). Psoriasis and Comorbid Diseases: Implications for Management. J. Am. Acad. Dermatol..

[14] AlQassimi S., AlBrashdi S., Galadari H., Hashim M.J. (2020). Global Burden of Psoriasis-Comparison of Regional and Global Epidemiology, 1990 to 2017. Int. J. Dermatol..

[15] Parisi R., Iskandar I.Y.K., Kontopantelis E., Augustin M., Griffiths C.E.M., Ashcroft D.M. (2020). National, Regional, and Worldwide Epidemiology of Psoriasis: Systematic Analysis and Modelling Study. BMJ.

[16] Michalek I.M., Loring B., John S.M. (2017). A Systematic Review of Worldwide Epidemiology of Psoriasis. J. Eur. Acad. Dermatol. Venereol..

[17] Raychaudhuri S.K., Maverakis E., Raychaudhuri S.P. (2014). Diagnosis and Classification of Psoriasis. Autoimmun. Rev..

[18] Ogawa E., Okuyama R., Seki T., Kobayashi A., Oiso N., Muto M., Nakagawa H., Kawada A. (2018). Epidemiological Survey of Patients with Psoriasis in Matsumoto City, Nagano Prefecture, Japan. J. Dermatol..

[19] Boehncke W.-H., Schön M.P. (2015). Psoriasis. Lancet.

[20] Zhang H., Hou W., Henrot L., Schnebert S., Dumas M., Heusèle C., Yang J. (2015). Modelling *Epidermis homoeostasis* and *Psoriasis pathogenesis*. J. R. Soc. Interface.

[21] Kim S.A., Ryu Y.W., Kwon J.I., Choe M.S., Jung J.W., Cho J.W. (2018). Differential Expression of Cyclin D1, Ki-67, pRb, and p53 in Psoriatic Skin Lesions and Normal Skin. Mol. Med. Report..

[22] Hwang Y.-J., Na J.-I., Byun S.-Y., Kwon S.-H., Yang S.-H., Lee H.-S., Choi H.-R., Cho S., Youn S.W., Park K.-C. (2020). Histone Deacetylase 1 and Sirtuin 1 Expression in Psoriatic Skin: A Comparison between Guttate and Plaque Psoriasis. Life.

[23] Zhang X., Yin M., Zhang L.-j. (2019). Keratin 6, 16 and 17-Critical Barrier Alarmin Molecules in Skin Wounds and Psoriasis. Cells.

[24] Ota T., Takekoshi S., Takagi T., Kitatani K., Toriumi K., Kojima T., Kato M., Ikoma N., Mabuchi T., Ozawa A. (2014). Notch Signaling May be Involved in the Abnormal Differentiation of Epidermal Keratinocytes in Psoriasis. Acta Histochem. Cytochem..

[25] Fu M., Wang G. (2012). Keratin 17 as a Therapeutic Target for the Treatment of Psoriasis. J. Dermatol. Sci..

[26] Elango T., Sun J., Zhu C., Zhou F., Zhang Y., Sun L., Yang S., Zhang X. (2018). Mutational Analysis of Epidermal and Hyperproliferative Type I Keratins in Mild and Moderate *Psoriasis vulgaris* Patients: A Possible Role in the Pathogenesis of Psoriasis along with Disease Severity. Hum. Genom..

[27] Akhlaghi M., Karrabi M., Atabti H., Raoofi A., Mousavi Khaneghah A. (2021). Investigation of the Role of IL18, IL-1β and NLRP3 Inflammasome in Reducing Expression of FLG-2 Protein in *Psoriasis vulgaris* Skin Lesions. Biotech. Histochem..

[28] Kim B.E., Howell M.D., Guttman E., Gilleaudeau P.M., Cardinale I.R., Boguniewicz M., Krueger J.G., Leung D.Y.M. (2011). TNF-a; Downregulates Filaggrin and Loricrin through c-Jun N-terminal Kinase: Role for TNF-a; Antagonists to Improve Skin Barrier. J. Investig. Dermatol..

[29] Lowes M.A., Bowcock A.M., Krueger J.G. (2007). Pathogenesis and Therapy of Psoriasis. Nature.

[30] Blauvelt A., Chiricozzi A. (2018). The Immunologic Role of IL-17 in Psoriasis and Psoriatic Arthritis Pathogenesis. Clin. Rev. Allergy Immunol..

[31] Furue M., Furue K., Tsuji G., Nakahara T. (2020). Interleukin-17A and Keratinocytes in Psoriasis. Int. J. Mol. Sci..

[32] Chen X., Cai G., Liu C., Zhao J., Gu C., Wu L., Hamilton T.A., Zhang C.-J., Ko J., Zhu L. (2019). IL-17R-EGFR Axis Links Wound Healing to Tumorigenesis in Lrig1(+) Stem Cells. J. Exp. Med..

[33] Wang X., Liu X., Liu N., Chen H. (2020). Prediction of Crucial Epigenetically-Associated, Differentially Expressed Genes by Integrated Bioinformatics Analysis and the Identification of S100A9 as a Novel Biomarker in Psoriasis. Int. J. Mol. Med..

[34] Zhuang L., Ma W., Yan J., Zhong H. (2020). Evaluation of the Effects of IL-22 on the Proliferation and Differentiation of Keratinocytes In Vitro. Mol. Med. Rep..

[35] Ekman A.-K., Bivik Eding C., Rundquist I., Enerbäck C. (2019). IL-17 and IL-22 Promote Keratinocyte Stemness in the Germinative Compartment in Psoriasis. J. Investig. Dermatol..

[36] Piro M.C., Ventura A., Smirnov A., Saggini A., Lena A.M., Mauriello A., Bianchi L., Melino G., Candi E. (2020). Transglutaminase 3 Reduces the Severity of Psoriasis in Imiquimod-Treated Mouse Skin. Int. J. Mol. Sci..

[37] Matsuki M., Yamashita F., Ishida-Yamamoto A., Yamada K., Kinoshita C., Fushiki S., Ueda E., Morishima Y., Tabata K., Yasuno H. (1998). Defective *Stratum corneum* and Early Neonatal Death in Mice Lacking the Gene for Transglutaminase 1 (Keratinocyte Transglutaminase). Proc. Natl. Acad. Sci. USA.

[38] Peltonen S., Riehokainen J., Pummi K., Peltonen J. (2007). Tight Junction Components Occludin, ZO-1, and Claudin-1, -4 and -5 in Active and Healing Psoriasis. Br. J. Dermatol..

[39] Kirschner N., Poetzl C., von den Driesch P., Wladykowski E., Moll I., Behne M.J., Brandner J.M. (2009). Alteration of Tight Junction Proteins is an Early Event in Psoriasis: Putative Involvement of Proinflammatory Cytokines. Am. J. Pathol..

[40] Visconti B., Paolino G., Carotti S., Pendolino A.L., Morini S., Richetta A.G., Calvieri S. (2015). Immunohistochemical Expression of VDR is Associated with Reduced Integrity of Tight Junction Complex in Psoriatic Skin. J. Eur. Acad. Dermatol. Venereol..

[41] Montero-Vilchez T., Segura-Fernández-Nogueras M.-V., Pérez-Rodríguez I., Soler-Gongora M., Martinez-Lopez A., Fernández-González A., Molina-Leyva A., Arias-Santiago S. (2021). Skin Barrier Function in Psoriasis and Atopic Dermatitis: Transepidermal Water Loss and Temperature as Useful Tools to Assess Disease Severity. J. Clin. Med..

[42] Watson R.E.B., Poddar R., Walker J.M., McGuill I., Hoare L.M., Griffiths C.E.M., O’Neill C.A. (2007). Altered Claudin Expression is a Feature of Chronic Plaque Psoriasis. J. Pathol..

[43] Gutowska-Owsiak D., Schaupp A.L., Salimi M., Selvakumar T.A., McPherson T., Taylor S., Ogg G.S. (2012). IL-17 Downregulates Filaggrin and Affects Keratinocyte Expression of Genes Associated with Cellular Adhesion. Exp. Dermatol..

[44] Chung E., Cook P.W., Parkos C.A., Park Y.-K., Pittelkow M.R., Coffey R.J. (2005). Amphiregulin Causes Functional Downregulation of Adherens Junctions in Psoriasis. J. Investig. Dermatol..

[45] Allen M., Ishida-Yamamoto A., McGrath J., Davison S., Iizuka H., Simon M., Guerrin M., Hayday A., Vaughan R., Serre G. (2001). Corneodesmosin Expression in Psoriasis Vulgaris Differs from Normal Skin and Other Inflammatory Skin Disorders. Lab. Investig..

[46] Simon M., Tazi-Ahnini R., Jonca N., Caubet C., Cork M.J., Serre G. (2008). Alterations in the Desquamation-Related Proteolytic Cleavage of Corneodesmosin and other Corneodesmosomal Proteins in Psoriatic Lesional Epidermis. Br. J. Dermatol..

[47] Labarthe M.-P., Saurat J.-H., Salomon D., Bosco D., Meda P. (1998). Upregulation of Connexin 26 between Keratinocytes of Psoriatic Lesions. J. Investig. Dermatol..

[48] Stylianaki E.-A., Karpouzis A., Tripsianis G., Veletza S. (2019). Assessment of Gap Junction Protein Beta-2 rs3751385 Gene Polymorphism in Psoriasis Vulgaris. J. Clin. Med. Res..

[49] Sun L.-D., Cheng H., Wang Z.-X., Zhang A.-P., Wang P.-G., Xu J.-H., Zhu Q.-X., Zhou H.-S., Ellinghaus E., Zhang F.-R. (2010). Association Analyses Identify Six New Psoriasis Susceptibility Loci in the Chinese Population. Nat. Genet..

[50] O’Shaughnessy E.M., Duffy W., Garcia-Vega L., Hussey K., Burden A.D., Zamiri M., Martin P.E. (2021). Dysregulation of Connexin Expression Plays a Pivotal Role in Psoriasis. Int. J. Mol. Sci..

[51] Yokose U., Ishikawa J., Morokuma Y., Naoe A., Inoue Y., Yasuda Y., Tsujimura H., Fujimura T., Murase T., Hatamochi A. (2020). The Ceramide [NP]/[NS] Ratio in the *Stratum corneum* is a Potential Marker for Skin Properties and Epidermal Differentiation. BMC Dermatol..

[52] Łuczaj W., Wroński A., Domingues P., Domingues M.R., Skrzydlewska E. (2020). Lipidomic Analysis Reveals Specific Differences between Fibroblast and Keratinocyte Ceramide Profile of Patients with Psoriasis Vulgaris. Molecules.

[53] Nakajima K., Terao M., Takaishi M., Kataoka S., Goto-Inoue N., Setou M., Horie K., Sakamoto F., Ito M., Azukizawa H. (2013). Barrier Abnormality Due to Ceramide Deficiency Leads to Psoriasiform Inflammation in a Mouse Model. J. Investig. Dermatol..

[54] Pietrzak A., Michalak-Stoma A., Chodorowska G., Szepietowski J.C. (2010). Lipid Disturbances in Psoriasis: An Update. Mediators Inflamm..

[55] Varshney P., Narasimhan A., Mittal S., Malik G., Sardana K., Saini N. (2016). Transcriptome Profiling Unveils the Role of Cholesterol in IL-17A Signaling in Psoriasis. Sci. Rep..

[56] Knox S., O’Boyle N.M. (2021). Skin Lipids in Health and Disease: A Review. Chem. Phys. Lipids.

[57] Farwanah H., Raith K., Neubert R.H.H., Wohlrab J. (2005). Ceramide Profiles of the Uninvolved Skin in Atopic Dermatitis and Psoriasis are Comparable to Those of Healthy Skin. Arch. Dermatol. Res..

[58] Patel R., Kevin Heard L., Chen X., Bollag W.B., Yang B. (2017). Aquaporins in the Skin. Aquaporins.

[59] Voss K.E., Bollag R.J., Fussell N., By C., Sheehan D.J., Bollag W.B. (2011). Abnormal Aquaporin-3 Protein Expression in Hyperproliferative Skin Disorders. Arch. Dermatol. Res..

[60] Ramadan W., Gheida S., El-Ashmawy A., Shareef M. (2017). Aquaporin-3 Expression in Common Hyperproliferative Skin Disorders: An Immunohistochemical Study. J. Egypt. Women’s Dermatol. Soc..

[61] Lee Y., Je Y.-J., Lee S.-S., Li Z.J., Choi D.-K., Kwon Y.-B., Sohn K.-C., Im M., Seo Y.J., Lee J.H. (2012). Changes in Transepidermal Water Loss and Skin Hydration According to Expression of Aquaporin-3 in Psoriasis. Ann. Dermatol..

[62] Krueger G.G., Jorgensen C.M. (1990). Experimental Models for Psoriasis. J. Investig. Dermatol..

[63] Barygina V., Becatti M., Prignano F., Lotti T., Taddei N., Fiorillo C. (2019). Fibroblasts to Keratinocytes Redox Signaling: The Possible Role of ROS in Psoriatic Plaque Formation. Antioxidants.

[64] Iwata H., Haga N., Ujiie H. (2021). Possible Role of Epiregulin from Dermal Fibroblasts in the Keratinocyte Hyperproliferation of Psoriasis. J. Dermatol..

[65] Gęgotek A., Domingues P., Wroński A., Skrzydlewska E. (2020). Changes in Proteome of Fibroblasts Isolated from Psoriatic Skin Lesions. Int. J. Mol. Sci..

[66] Ten Bergen L.L., Petrovic A., Aarebrot A.K., Appel S. (2020). Current Knowledge on Autoantigens and Autoantibodies in Psoriasis. Scand. J. Immunol..

[67] Lou F., Sun Y., Xu Z., Niu L., Wang Z., Deng S., Liu Z., Zhou H., Bai J., Yin Q. (2020). Excessive Polyamine Generation in Keratinocytes Promotes Self-RNA Sensing by Dendritic Cells in Psoriasis. Immunity.

[68] Méndez-Samperio P. (2010). The Human Cathelicidin hCAP18/LL-37: A Multifunctional Peptide Involved in Mycobacterial Infections. Peptides.

[69] Lande R., Gregorio J., Facchinetti V., Chatterjee B., Wang Y.-H., Homey B., Cao W., Wang Y.-H., Su B., Nestle F.O. (2007). Plasmacytoid Dendritic Cells Sense Self-DNA Coupled with Antimicrobial Peptide. Nature.

[70] Ganguly D., Chamilos G., Lande R., Gregorio J., Meller S., Facchinetti V., Homey B., Barrat F.J., Zal T., Gilliet M. (2009). Self-RNA-Antimicrobial Peptide Complexes Activate Human Dendritic Cells through TLR7 and TLR8. J. Exp. Med..

[71] Morizane S., Yamasaki K., Mühleisen B., Kotol P.F., Murakami M., Aoyama Y., Iwatsuki K., Hata T., Gallo R.L. (2012). Cathelicidin Antimicrobial Peptide LL-37 in Psoriasis Enables Keratinocyte Reactivity against TLR9 Ligands. J. Investig. Dermatol..

[72] Lande R., Botti E., Jandus C., Dojcinovic D., Fanelli G., Conrad C., Chamilos G., Feldmeyer L., Marinari B., Chon S. (2014). The Antimicrobial Peptide LL37 is a T-Cell Autoantigen in Psoriasis. Nat. Commun..

[73] Bader H.L., Wang L.W., Ho J.C., Tran T., Holden P., Fitzgerald J., Atit R.P., Reinhardt D.P., Apte S.S. (2012). A Disintegrin-Like and Metalloprotease Domain Containing Thrombospondin Type 1 Motif-Like 5 (ADAMTSL5) is a Novel Fibrillin-1-, Fibrillin-2-, and Heparin-Binding Member of the ADAMTS Superfamily Containing a Netrin-Like Module. Matrix Biol..

[74] Arakawa A., Siewert K., Stöhr J., Besgen P., Kim S.-M., Rühl G., Nickel J., Vollmer S., Thomas P., Krebs S. (2015). Melanocyte Antigen Triggers Autoimmunity in Human Psoriasis. J. Exp. Med..

[75] Chiba H., Michibata H., Wakimoto K., Seishima M., Kawasaki S., Okubo K., Mitsui H., Torii H., Imai Y. (2004). Cloning of a Gene for a Novel Epithelium-Specific Cytosolic Phospholipase A2, cPLA2δ, Induced in Psoriatic Skin. J. Biol. Chem..

[76] Cheung K.L., Jarrett R., Subramaniam S., Salimi M., Gutowska-Owsiak D., Chen Y.-L., Hardman C., Xue L., Cerundolo V., Ogg G. (2016). Psoriatic T Cells Recognize Neolipid Antigens Generated by Mast Cell Phospholipase Delivered by Exosomes and Presented by CD1a. J. Exp. Med..

[77] Bragulla H.H., Homberger D.G. (2009). Structure and Functions of Keratin Proteins in Simple, Stratified, Keratinized and Cornified Epithelia. J. Anat..

[78] Johnston A., Gudjonsson J.E., Sigmundsdottir H., Love T.J., Valdimarsson H. (2004). Peripheral Blood T Cell Responses to Keratin Peptides that Share Sequences with Streptococcal M Proteins are Largely Restricted to Skin-Homing CD8(+) T Cells. Clin. Exp. Immunol..

[79] Besgen P., Trommler P., Vollmer S., Prinz J.C. (2010). Ezrin, Maspin, Peroxiredoxin 2, and Heat Shock Protein 27: Potential Targets of a Streptococcal-Induced Autoimmune Response in Psoriasis. J. Immunol..

[80] Jean-Philippe J., Paz S., Caputi M. (2013). hnRNP A1: The Swiss Army Knife of Gene Expression. Int. J. Mol. Sci..

[81] Guarneri C., Aguennouz M., Guarneri F., Polito F., Benvenga S., Cannavò S.P. (2018). Autoimmunity to Heterogeneous Nuclear Ribonucleoprotein A1 in Psoriatic Patients and Correlation with Disease Severity. J. Dtsch. Dermatol. Ges..

[82] Niyonsaba F., Ogawa H. (2005). Protective Roles of the Skin against Infection: Implication of Naturally Occurring Human Antimicrobial Agents β-Defensins, Cathelicidin LL-37 and Lysozyme. J. Dermatol. Sci..

[83] Lande R., Chamilos G., Ganguly D., Demaria O., Frasca L., Durr S., Conrad C., Schröder J., Gilliet M. (2015). Cationic antimicrobial Peptides in Psoriatic Skin Cooperate to Break Innate Tolerance to Self-DNA. Eur. J. Immunol..

[84] Takahashi T., Yamasaki K. (2020). Psoriasis and Antimicrobial Peptides. Int. J. Mol. Sci..

[85] Albanesi C., Madonna S., Gisondi P., Girolomoni G. (2018). The Interplay between Keratinocytes and Immune Cells in the Pathogenesis of Psoriasis. Front. Immunol..

[86] Cai Y., Xue F., Quan C., Qu M., Liu N., Zhang Y., Fleming C., Hu X., Zhang H.-G., Weichselbaum R. (2019). A Critical Role of the IL-1β-IL-1R Signaling Pathway in Skin Inflammation and Psoriasis Pathogenesis. J. Investig. Dermatol..

[87] Jiang M., Fang H., Shao S., Dang E., Zhang J., Qiao P., Yang A., Wang G. (2019). Keratinocyte Exosomes Activate Neutrophils and Enhance Skin Inflammation in Psoriasis. FASEB J..

[88] Orlik C., Deibel D., Küblbeck J., Balta E., Ganskih S., Habicht J., Niesler B., Schröder-Braunstein J., Schäkel K., Wabnitz G. (2020). Keratinocytes Costimulate Naive Human T Cells via CD2: A Potential Target to Prevent the Development of Proinflammatory Th1 Cells in the Skin. Cell. Mol. Immunol..

[89] Albanesi C., Scarponi C., Pallotta S., Daniele R., Bosisio D., Madonna S., Fortugno P., Gonzalvo-Feo S., Franssen J.-D., Parmentier M. (2009). Chemerin Expression Marks Early Psoriatic Skin Lesions and Correlates with Plasmacytoid Dendritic Cell Recruitment. J. Exp. Med..

[90] Tohyama M., Yang L., Hanakawa Y., Dai X., Shirakata Y., Sayama K. (2012). IFN-α Enhances IL-22 Receptor Expression in Keratinocytes: A Possible Role in the Development of Psoriasis. J. Investig. Dermatol..

[91] O’Keeffe M., Mok W.H., Radford K.J. (2015). Human Dendritic Cell Subsets and Function in Health and Disease. Cell. Mol. Life Sci..

[92] Kim T.-G., Jee H., Fuentes-Duculan J., Wu W.H., Byamba D., Kim D.-S., Kim D.-Y., Lew D.-H., Yang W.-I., Krueger J.G. (2014). Dermal Clusters of Mature Dendritic Cells and T Cells Are Associated with the CCL20/CCR6 Chemokine System in Chronic Psoriasis. J. Investig. Dermatol..

[93] Chiricozzi A., Romanelli P., Volpe E., Borsellino G., Romanelli M. (2018). Scanning the Immunopathogenesis of Psoriasis. Int. J. Mol. Sci..

[94] Terhorst D., Chelbi R., Wohn C., Malosse C., Tamoutounour S., Jorquera A., Bajenoff M., Dalod M., Malissen B., Henri S. (2015). Dynamics and Transcriptomics of Skin Dendritic Cells and Macrophages in an Imiquimod-Induced, Biphasic Mouse Model of Psoriasis. J. Immunol..

[95] Glitzner E., Korosec A., Brunner P.M., Drobits B., Amberg N., Schonthaler H.B., Kopp T., Wagner E.F., Stingl G., Holcmann M. (2014). Specific Roles for Dendritic Cell Subsets during Initiation and Progression of Psoriasis. EMBO Mol. Med..

[96] Lee M., Kim S.H., Kim T.-G., Park J., Lee J.W., Lee M.-G. (2018). Resident and Monocyte-Derived Langerhans Cells are Required for Imiquimod-Induced Psoriasis-Like Dermatitis Model. J. Dermatol. Sci..

[97] Zheng T., Zhao W., Li H., Xiao S., Hu R., Han M., Liu H., Liu Y., Otsu K., Liu X. (2018). p38α Signaling in Langerhans cells Promotes the Development of IL-17–Producing T Cells and Psoriasiform Skin Inflammation. Sci. Signal..

[98] Singh T.P., Zhang H.H., Borek I., Wolf P., Hedrick M.N., Singh S.P., Kelsall B.L., Clausen B.E., Farber J.M. (2016). Monocyte-derived Inflammatory Langerhans Cells and Dermal Dendritic Cells Mediate Psoriasis-Like Inflammation. Nat. Commun..

[99] Fanoni D., Venegoni L., Vergani B., Tavecchio S., Cattaneo A., Leone B.E., Berti E., Marzano A.V. (2019). Evidence for a Role of Autoinflammation in Early-Phase Psoriasis. Clin. Exp. Immunol..

[100] Chiang C.-C., Cheng W.-J., Korinek M., Lin C.-Y., Hwang T.-L. (2019). Neutrophils in Psoriasis. Front. Immunol..

[101] Mutua V., Gershwin L.J. (2020). A Review of Neutrophil Extracellular Traps (NETs) in Disease: Potential Anti-NETs Therapeutics. Clin. Rev. Allergy Immunol..

[102] Lambert S., Hambro C.A., Johnston A., Stuart P.E., Tsoi L.C., Nair R.P., Elder J.T. (2019). Neutrophil Extracellular Traps Induce Human Th17 Cells: Effect of Psoriasis-Associated TRAF3IP2 Genotype. J. Investig. Dermatol..

[103] Herster F., Bittner Z., Archer N.K., Dickhöfer S., Eisel D., Eigenbrod T., Knorpp T., Schneiderhan-Marra N., Löffler M.W., Kalbacher H. (2020). Neutrophil Extracellular Trap-Associated RNA and LL37 Enable Self-Amplifying Inflammation in Psoriasis. Nat. Commun..

[104] Wang Y., Edelmayer R., Wetter J., Salte K., Gauvin D., Leys L., Paulsboe S., Su Z., Weinberg I., Namovic M. (2019). Monocytes/Macrophages Play a Pathogenic Role in IL-23 Mediated Psoriasis-Like Skin Inflammation. Sci. Rep..

[105] Yawalkar N., Karlen S., Hunger R., Brand C.U., Braathen L.R. (1998). Expression of Interleukin-12 is Increased in Psoriatic Skin. J. Investig. Dermatol..

[106] Fuentes-Duculan J., Suárez-Fariñas M., Zaba L.C., Nograles K.E., Pierson K.C., Mitsui H., Pensabene C.A., Kzhyshkowska J., Krueger J.G., Lowes M.A. (2010). A Subpopulation of CD163-Positive Macrophages is Classically Activated in Psoriasis. J. Investig. Dermatol..

[107] Cai Y., Shen X., Ding C., Qi C., Li K., Li X., Jala V.R., Zhang H.-g., Wang T., Zheng J. (2011). Pivotal Role of Dermal IL-17-Producing γδ T Cells in Skin Inflammation. Immunity.

[108] Hou Y., Zhu L., Tian H., Sun H.-X., Wang R., Zhang L., Zhao Y. (2018). IL-23-Induced Macrophage Polarization and its Pathological Roles in Mice with Imiquimod-Induced Psoriasis. Protein Cell.

[109] Lin A.M., Rubin C.J., Khandpur R., Wang J.Y., Riblett M., Yalavarthi S., Villanueva E.C., Shah P., Kaplan M.J., Bruce A.T. (2011). Mast Cells and Neutrophils Release IL-17 through Extracellular Trap Formation in Psoriasis. J. Immunol..

[110] Mashiko S., Bouguermouh S., Rubio M., Baba N., Bissonnette R., Sarfati M. (2015). Human Mast Cells are Major IL-22 Producers in Patients with Psoriasis and Atopic Dermatitis. J. Allergy Clin. Immunol..

[111] Peres L.P., Oliveira F.B., Cartell A., Mazzotti N.G., Cestari T.F. (2018). Density of Mast Cells and Intensity of Pruritus in Psoriasis Vulgaris: A Cross Sectional Study. An. Bras. Dermatol..

[112] Keren A., Shemer A., Ginzburg A., Ullmann Y., Schrum A.G., Paus R., Gilhar A. (2018). Innate Lymphoid Cells 3 Induce Psoriasis in Xenotransplanted Healthy Human Skin. J. Allergy Clin. Immunol..

[113] Bielecki P., Riesenfeld S.J., Hütter J.-C., Torlai Triglia E., Kowalczyk M.S., Ricardo-Gonzalez R.R., Lian M., Amezcua Vesely M.C., Kroehling L., Xu H. (2021). Skin-Resident Innate Lymphoid Cells Converge on a Pathogenic Effector State. Nature.

[114] Teunissen M.B.M., Munneke J.M., Bernink J.H., Spuls P.I., Res P.C.M., te Velde A., Cheuk S., Brouwer M.W.D., Menting S.P., Eidsmo L. (2014). Composition of Innate Lymphoid Cell Subsets in the Human Skin: Enrichment of NCR + ILC3 in Lesional Skin and Blood of Psoriasis Patients. J. Investig. Dermatol..

[115] Sato Y., Ogawa E., Okuyama R. (2020). Role of Innate Immune Cells in Psoriasis. Int. J. Mol. Sci..

[116] Polese B., Zhang H., Thurairajah B., King I.L. (2020). Innate Lymphocytes in Psoriasis. Front. Immunol..

[117] Laggner U., Di Meglio P., Perera G.K., Hundhausen C., Lacy K.E., Ali N., Smith C.H., Hayday A.C., Nickoloff B.J., Nestle F.O. (2011). Identification of a Novel Proinflammatory Human Skin-Homing Vγ9Vδ2 T Cell Subset with a Potential Role in Psoriasis. J. Immunol..

[118] Plužarić V., Štefanić M., Mihalj M., Tolušić Levak M., Muršić I., Glavaš-Obrovac L., Petrek M., Balogh P., Tokić S. (2020). Differential Skewing of Circulating MR1-Restricted and γδ T Cells in Human Psoriasis Vulgaris. Front. Immunol..

[119] Ramírez-Valle F., Gray E.E., Cyster J.G. (2015). Inflammation Induces Dermal Vγ4+ γδT17 Memory-Like Cells that Travel to Distant Skin and Accelerate Secondary IL-17-Driven Responses. Proc. Natl. Acad. Sci. USA.

[120] Hartwig T., Pantelyushin S., Croxford A.L., Kulig P., Becher B. (2015). Dermal IL-17-Producing γδ T Cells Establish Long-Lived Memory in the Skin. Eur. J. Immunol..

[121] Sumaria N., Roediger B., Ng L.G., Qin J., Pinto R., Cavanagh L.L., Shklovskaya E., Fazekas de St Groth B., Triccas J.A., Weninger W. (2011). Cutaneous Immunosurveillance by Self-Renewing Dermal Gammadelta T Cells. J. Exp. Med..

[122] Bos J.D., Teunissen M.B.M., Cairo I., Krieg S.R., Kapsenberg M.L., Das P.K., Borst J. (1990). T-Cell Receptor γδ Bearing Cells in Normal Human Skin. J. Investig. Dermatol..

[123] Ebert L.M., Meuter S., Moser B. (2006). Homing and Function of Human Skin γδ T Cells and NK Cells: Relevance for Tumor Surveillance. J. Immunol..

[124] Zhu X., Zhu J. (2020). CD4 T Helper Cell Subsets and Related Human Immunological Disorders. Int. J. Mol. Sci..

[125] Raphael I., Nalawade S., Eagar T.N., Forsthuber T.G. (2015). T Cell Subsets and Their Signature Cytokines in Autoimmune and Inflammatory Diseases. Cytokine.

[126] Austin L.M., Ozawa M., Kikuchi T., Walters I.B., Krueger J.G. (1999). The Majority of Epidermal T Cells in Psoriasis Vulgaris Lesions can Produce Type 1 Cytokines, Interferon-γ, Interleukin-2, and Tumor Necrosis Factor-α, Defining TC1 (Cytotoxic T Lymphocyte) and TH1 Effector Populations: A Type 1 Differentiation Bias is also Measured in Circulating Blood T Cells in Psoriatic Patients. J. Investig. Dermatol..

[127] Furiati S.C., Catarino J.S., Silva M.V., Silva R.F., Estevam R.B., Teodoro R.B., Pereira S.L., Ataide M., Rodrigues V., Rodrigues D.B.R. (2019). Th1, Th17, and Treg Responses are Differently Modulated by TNF-α Inhibitors and Methotrexate in Psoriasis Patients. Sci. Rep..

[128] Reich K., Reich J.L.K., Falk T.M., Blödorn-Schlicht N., Mrowietz U., von Kiedrowski R., Pfeiffer C., Niesmann J., Frambach Y., Warren R.B. (2019). Clinical Response of Psoriasis to Subcutaneous Methotrexate Correlates with Inhibition of Cutaneous T Helper 1 and 17 Inflammatory Pathways. Br. J. Dermatol..

[129] Priyadarssini M., Chandrashekar L., Rajappa M. (2019). Effect of Methotrexate Monotherapy on T-Cell Subsets in the Peripheral Circulation in Psoriasis. Clin. Exp. Dermatol..

[130] Chiricozzi A., Guttman-Yassky E., Suárez-Fariñas M., Nograles K.E., Tian S., Cardinale I., Chimenti S., Krueger J.G. (2011). Integrative Responses to IL-17 and TNF-α in Human Keratinocytes Account for Key Inflammatory Pathogenic Circuits in Psoriasis. J. Investig. Dermatol..

[131] Nograles K.E., Zaba L.C., Guttman-Yassky E., Fuentes-Duculan J., Suárez-Fariñas M., Cardinale I., Khatcherian A., Gonzalez J., Pierson K.C., White T.R. (2008). Th17 Cytokines Interleukin (IL)-17 and IL-22 Modulate Distinct Inflammatory and Keratinocyte-Response Pathways. Br. J. Dermatol..

[132] Wilson N.J., Boniface K., Chan J.R., McKenzie B.S., Blumenschein W.M., Mattson J.D., Basham B., Smith K., Chen T., Morel F. (2007). Development, Cytokine Profile and Function of Human Interleukin 17–Producing Helper T Cells. Nat. Immunol..

[133] Harper E.G., Guo C., Rizzo H., Lillis J.V., Kurtz S.E., Skorcheva I., Purdy D., Fitch E., Iordanov M., Blauvelt A. (2009). Th17 Cytokines Stimulate CCL20 Expression in Keratinocytes In Vitro and In Vivo: Implications for *Psoriasis pathogenesis*. J. Investig. Dermatol..

[134] Matos T.R., O’Malley J.T., Lowry E.L., Hamm D., Kirsch I.R., Robins H.S., Kupper T.S., Krueger J.G., Clark R.A. (2017). Clinically Resolved Psoriatic Lesions Contain Psoriasis-Specific IL-17-Producing αβ T Cell Clones. J. Clin. Investig..

[135] Hawkes J.E., Yan B.Y., Chan T.C., Krueger J.G. (2018). Discovery of the IL-23/IL-17 Signaling Pathway and the Treatment of Psoriasis. J. Immunol..

[136] Stockinger B., Omenetti S. (2017). The Dichotomous Nature of T Helper 17 Cells. Nat. Rev. Immunol..

[137] Aguilar-Flores C., Castro-Escamilla O., Ortega-Rocha E.M., Maldonado-García C., Jurado-Santa Cruz F., Pérez-Montesinos G., Lemini-López A., Bonifaz L.C. (2020). Association of Pathogenic Th17 Cells with the Disease Severity and Its Potential Implication for Biological Treatment Selection in Psoriasis Patients. Mediat. Inflamm..

[138] Wu L., Hollinshead K.E.R., Hao Y., Au C., Kroehling L., Ng C., Lin W.-Y., Li D., Silva H.M., Shin J. (2020). Niche-Selective Inhibition of Pathogenic Th17 Cells by Targeting Metabolic Redundancy. Cell.

[139] Ortega C., Fernández A.S., Carrillo J.M., Romero P., Molina I.J., Moreno J.C., Santamaría M. (2009). IL-17-Producing CD8+ T Lymphocytes from Psoriasis Skin Plaques are Cytotoxic Effector Cells that Secrete Th17-Related Cytokines. J. Leukoc. Biol..

[140] Hijnen D., Knol E.F., Gent Y.Y., Giovannone B., Beijn S.J.P., Kupper T.S., Bruijnzeel-Koomen C.A.F.M., Clark R.A. (2013). CD8(+) T Cells in the Lesional Skin of Atopic Dermatitis and Psoriasis Patients Are an Important Source of IFN-γ, IL-13, IL-17, and IL-22. J. Investig. Dermatol..

[141] Vičić M., Kaštelan M., Sotošek Tokmadžić V., Prpić Massari L. (2019). Systemic and Local Increase of Granulysin Expression in Cytotoxic Lymphocytes in Severe Psoriasis. Acta Derm. Venereol..

[142] Liu J., Chang H.-W., Huang Z.-M., Nakamura M., Sekhon S., Ahn R., Munoz-Sandoval P., Bhattarai S., Beck K.M., Sanchez I.M. (2021). Single-Cell RNA Sequencing of Psoriatic Skin Identifies Pathogenic Tc17 Cell Subsets and Reveals Distinctions between CD8+ T Cells in Autoimmunity and Cancer. J. Allergy Clin. Immunol..

[143] Watanabe R. (2019). Protective and Pathogenic Roles of Resident Memory T Cells in Human Skin Disorders. J. Dermatol. Sci..

[144] Kurihara K., Fujiyama T., Phadungsaksawasdi P., Ito T., Tokura Y. (2019). Significance of IL-17A-Producing CD8+CD103+ Skin Resident Memory T Cells in Psoriasis Lesion and Their Possible Relationship to Clinical Course. J. Dermatol. Sci..

[145] Choi C.W., Kim B.R., Yang S., Kim Y., Kang J.S., Youn S.W. (2020). Regulatory T Cells Suppress Skin Inflammation in the Imiquimod-Induced Psoriasis-Like Mouse Model. J. Dermatol. Sci..

[146] Stockenhuber K., Hegazy A.N., West N.R., Ilott N.E., Stockenhuber A., Bullers S.J., Thornton E.E., Arnold I.C., Tucci A., Waldmann H. (2018). Foxp3(+) T Reg Cells Control Psoriasiform Inflammation by Restraining an IFN-I-Driven CD8(+) T Cell Response. J. Exp. Med..

[147] Hau C.S., Shimizu T., Tada Y., Kamata M., Takeoka S., Shibata S., Mitsui A., Asano Y., Sugaya M., Kadono T. (2018). The Vitamin D3 Analog, Maxacalcitol, Reduces Psoriasiform Skin Inflammation by Inducing Regulatory T Cells and Downregulating IL-23 and IL-17 Production. J. Dermatol. Sci..

[148] Sulaimani J., Cluxton D., Clowry J., Petrasca A., Molloy O.E., Moran B., Sweeney C.M., Malara A., McNicholas N., McGuigan C. (2021). Dimethyl Fumarate Modulates the Treg–Th17 Cell Axis in Patients with Psoriasis. Br. J. Dermatol..

[149] Nussbaum L., Chen Y.L., Ogg G.S. (2021). Role of Regulatory T Cells in Psoriasis Pathogenesis and Treatment. Br. J. Dermatol..

[150] Sugiyama H., Gyulai R., Toichi E., Garaczi E., Shimada S., Stevens S.R., McCormick T.S., Cooper K.D. (2005). Dysfunctional Blood and Target Tissue CD4 + CD25 High Regulatory T Cells in Psoriasis: Mechanism Underlying Unrestrained Pathogenic Effector T Cell Proliferation. J. Immunol..

[151] Li B., Lei J., Yang L., Gao C., Dang E., Cao T., Xue K., Zhuang Y., Shao S., Zhi D. (2019). Dysregulation of Akt-FOXO1 Pathway Leads to Dysfunction of Regulatory T Cells in Patients with Psoriasis. J. Investig. Dermatol..

[152] Goodman W.A., Levine A.D., Massari J.V., Sugiyama H., McCormick T.S., Cooper K.D. (2009). IL-6 Signaling in Psoriasis Prevents Immune Suppression by Regulatory T Cells. J. Immunol..

[153] Shi Y., Chen Z., Zhao Z., Yu Y., Fan H., Xu X., Bu X., Gu J. (2019). IL-21 Induces an Imbalance of Th17/Treg Cells in Moderate-to-Severe Plaque Psoriasis Patients. Front. Immunol..

[154] Yang L., Li B., Dang E., Jin L., Fan X., Wang G. (2016). Impaired Function of Regulatory T Cells in Patients with Psoriasis is Mediated by Phosphorylation of STAT3. J. Dermatol. Sci..

[155] Grän F., Kerstan A., Serfling E., Goebeler M., Muhammad K. (2020). Current Developments in the Immunology of Psoriasis. Yale J. Biol. Med..

[156] Hayashi M., Yanaba K., Umezawa Y., Yoshihara Y., Kikuchi S., Ishiuji Y., Saeki H., Nakagawa H. (2016). IL-10-Producing Regulatory B Cells Are Decreased in Patients with Psoriasis. J. Dermatol. Sci..

[157] Mizumaki K., Horii M., Kano M., Komuro A., Matsushita T. (2021). Suppression of IL-23-Mediated Psoriasis-Like Inflammation by Regulatory B Cells. Sci. Rep..

[158] Yanaba K., Kamata M., Ishiura N., Shibata S., Asano Y., Tada Y., Sugaya M., Kadono T., Tedder T.F., Sato S. (2013). Regulatory B Cells Suppress Imiquimod-Induced, Psoriasis-Like Skin Inflammation. J. Leukoc. Biol..

[159] Chen C., Tan L., Zhu W., Lei L., Kuang Y., Liu P., Li J., Chen X., Peng C. (2020). Targeting Myeloid-Derived Suppressor Cells Is a Novel Strategy for Anti-Psoriasis Therapy. Mediat. Inflamm..

[160] Ilkovitch D., Ferris L.K. (2016). Myeloid-Derived Suppressor Cells Are Elevated in Patients with Psoriasis and Produce Various Molecules. Mol. Med. Report..

[161] Cao L.Y., Chung J.-S., Teshima T., Feigenbaum L., Cruz P.D., Jacobe H.T., Chong B.F., Ariizumi K. (2016). Myeloid-Derived Suppressor Cells in Psoriasis Are an Expanded Population Exhibiting Diverse T-Cell-Suppressor Mechanisms. J. Investig. Dermatol..

[162] Kim C.-H., Yoo J.K., Jeon S.H., Lim C.-Y., Lee J.-H., Koo D.-B., Park M.-Y. (2019). Anti-Psoriatic Effect of Myeloid-Derived Suppressor Cells on Imiquimod-Induced Skin Inflammation in Mice. Scand. J. Immunol..

[163] Afonina I.S., Van Nuffel E., Beyaert R. (2021). Immune Responses and Therapeutic Options in Psoriasis. Cell. Mol. Life Sci..

[164] Wong V.W., Akaishi S., Longaker M.T., Gurtner G.C. (2011). Pushing Back: Wound Mechanotransduction in Repair and Regeneration. J. Investig. Dermatol..

[165] Lønnberg A.S., Skov L., Skytthe A., Kyvik K.O., Pedersen O.B., Thomsen S.F. (2013). Heritability of Psoriasis in a Large Twin Sample. Br. J. Dermatol..

[166] Grjibovski A.M., Olsen A.O., Magnus P., Harris J.R. (2007). Psoriasis in Norwegian Twins: Contribution of Genetic and Environmental Effects. J. Eur. Acad. Dermatol. Venereol..

[167] Ogawa K., Okada Y. (2020). The Current Landscape of Psoriasis Genetics in 2020. J. Dermatol. Sci..

[168] Gudjonsson J.E., Karason A., Hjaltey Runarsdottir E., Antonsdottir A.A., Hauksson V.B., Jónsson H.H., Gulcher J., Stefansson K., Valdimarsson H. (2006). Distinct Clinical Differences between HLA-Cw*0602 Positive and Negative Psoriasis Patients-An Analysis of 1019 HLA-C-And HLA-B-Typed Patients. J. Investig. Dermatol..

[169] Gudjonsson J.E., Thorarinsson A.M., Sigurgeirsson B., Kristinsson K.G., Valdimarsson H. (2003). Streptococcal Throat Infections and Exacerbation of Chronic Plaque Psoriasis: A Prospective Study. Br. J. Dermatol..

[170] Prinz J.C. (2017). Melanocytes: Target Cells of an HLA-C*06:02–Restricted Autoimmune Response in Psoriasis. J. Investig. Dermatol..

[171] Mabuchi T., Hirayama N. (2016). Binding Affinity and Interaction of LL-37 with HLA-C*06:02 in Psoriasis. J. Investig. Dermatol..

[172] Wei P., Yang Y., Liu Z., Luo Z., Tu W., Han J., Deng Y., Yin L. (2017). Characterization of Autoantigen Presentation by HLA-C*06:02 in Psoriasis. J. Investig. Dermatol..

[173] Ray-Jones H., Duffus K., McGovern A., Martin P., Shi C., Hankinson J., Gough O., Yarwood A., Morris A.P., Adamson A. (2020). Mapping DNA Interaction Landscapes in Psoriasis Susceptibility Loci Highlights KLF4 as a Target Gene in 9q31. BMC Biol..

[174] Segre J.A., Bauer C., Fuchs E. (1999). Klf4 is a Transcription Factor Required for Establishing the Barrier Function of the Skin. Nat. Genet..

[175] Zhang X.-J., Huang W., Yang S., Sun L.-D., Zhang F.-Y., Zhu Q.-X., Zhang F.-R., Zhang C., Du W.-H., Pu X.-M. (2009). Psoriasis Genome-Wide Association Study Identifies Susceptibility Variants within LCE Gene Cluster at 1q21. Nat. Genet..

[176] Yin X., Low H.Q., Wang L., Li Y., Ellinghaus E., Han J., Estivill X., Sun L., Zuo X., Shen C. (2015). Genome-Wide Meta-Analysis Identifies Multiple Novel Associations and Ethnic Heterogeneity of Psoriasis Susceptibility. Nat. Commun..

[177] Hirata J., Hirota T., Ozeki T., Kanai M., Sudo T., Tanaka T., Hizawa N., Nakagawa H., Sato S., Mushiroda T. (2018). Variants at HLA-A, HLA-C, and HLA-DQB1 Confer Risk of Psoriasis Vulgaris in Japanese. J. Investig. Dermatol..

[178] Van Vugt L.J., van den Reek J.M.P.A., Hannink G., Coenen M.J.H., de Jong E.M.G.J. (2019). Association of HLA-C*06:02 Status with Differential Response to Ustekinumab in Patients with Psoriasis: A Systematic Review and Meta-Analysis. JAMA Dermatol..

[179] Tsoi L.C., Stuart P.E., Tian C., Gudjonsson J.E., Das S., Zawistowski M., Ellinghaus E., Barker J.N., Chandran V., Dand N. (2017). Large Scale Meta-Analysis Characterizes Genetic Architecture for Common Psoriasis Associated Variants. Nat. Commun..

[180] Tsoi L.C., Spain S.L., Knight J., Ellinghaus E., Stuart P.E., Capon F., Ding J., Li Y., Tejasvi T., Gudjonsson J.E. (2012). Identification of 15 New Psoriasis Susceptibility Loci Highlights the Role of Innate Immunity. Nat. Genet..

[181] Goldminz A.M., Au S.C., Kim N., Gottlieb A.B., Lizzul P.F. (2013). NF-κB: An Essential Transcription Factor in Psoriasis. J. Dermatol. Sci..

[182] Tseng J.-C., Chang Y.-C., Huang C.-M., Hsu L.-C., Chuang T.-H. (2021). Therapeutic Development Based on the Immunopathogenic Mechanisms of Psoriasis. Pharmaceutics.

[183] Nair R.P., Duffin K.C., Helms C., Ding J., Stuart P.E., Goldgar D., Gudjonsson J.E., Li Y., Tejasvi T., Feng B.-J. (2009). Genome-Wide Scan Reveals Association of Psoriasis with IL-23 and NF-kappaB Pathways. Nat. Genet..

[184] Tokuyama M., Mabuchi T. (2020). New Treatment Addressing the Pathogenesis of Psoriasis. Int. J. Mol. Sci..

[185] Kvist-Hansen A., Hansen P.R., Skov L. (2020). Systemic Treatment of Psoriasis with JAK Inhibitors: A Review. Dermatol. Ther..

[186] Caputo V., Strafella C., Termine A., Campione E., Bianchi L., Novelli G., Giardina E., Cascella R. (2020). RNAseq-Based Prioritization Revealed COL6A5, COL8A1, COL10A1 and MIR146A as Common and Differential Susceptibility Biomarkers for Psoriasis and Psoriatic Arthritis: Confirmation from Genotyping Analysis of 1417 Italian Subjects. Int. J. Mol. Sci..

[187] Bergboer J.G.M., Tjabringa G.S., Kamsteeg M., van Vlijmen-Willems I.M.J.J., Rodijk-Olthuis D., Jansen P.A.M., Thuret J.-Y., Narita M., Ishida-Yamamoto A., Zeeuwen P.L.J.M. (2011). Psoriasis Risk Genes of the Late Cornified Envelope-3 Group Are Distinctly Expressed Compared with Genes of Other LCE Groups. Am. J. Pathol..

[188] Hoss E., Austin H.R., Batie S.F., Jurutka P.W., Haussler M.R., Whitfield G.K. (2013). Control of Late Cornified Envelope Genes Relevant to Psoriasis Risk: Upregulation by 1,25-Dihydroxyvitamin D3 and Plant-Derived Delphinidin. Arch. Dermatol. Res..

[189] Caputo V., Strafella C., Termine A., Dattola A., Mazzilli S., Lanna C., Cosio T., Campione E., Novelli G., Giardina E. (2020). Overview of the Molecular Determinants Contributing to the Expression of Psoriasis and Psoriatic Arthritis phenotypes. J. Cell. Mol. Med..

[190] Zhang P., Zhao M., Liang G., Yin G., Huang D., Su F., Zhai H., Wang L., Su Y., Lu Q. (2013). Whole-Genome DNA Methylation in Skin Lesions from Patients with Psoriasis Vulgaris. J. Autoimmun..

[191] Hu Y., Baud V., Oga T., Kim K.I., Yoshida K., Karin M. (2001). IKKα Controls Formation of the Epidermis Independently of NF-κB. Nature.

[192] Yu N., Yang Y., Li X., Zhang M., Huang J., Wang X., Long X. (2016). MiR-26a Inhibits Proliferation and Migration of HaCaT Keratinocytes through Regulating PTEN Expression. Gene.

[193] Verallo-Rowell V.M., Katalbas S.S., Evangelista M.T.P., Dayrit J.F. (2018). Review Update on Topical Therapy for Psoriasis. Curr. Dermatol. Rep..

[194] Bijlmakers M.-J., Kanneganti S.K., Barker J.N., Trembath R.C., Capon F. (2011). Functional Analysis of the RNF114 Psoriasis Susceptibility Gene Implicates Innate Immune Responses to Double-Stranded RNA in Disease Pathogenesis. Hum. Mol. Genet..

[195] Rácz E., Prens E.P., Kurek D., Kant M., de Ridder D., Mourits S., Baerveldt E.M., Ozgur Z., van Ijcken W.F.J., Laman J.D. (2011). Effective Treatment of Psoriasis with Narrow-Band UVB Phototherapy Is Linked to Suppression of the IFN and Th17 Pathways. J. Investig. Dermatol..

[196] Catapano M., Vergnano M., Romano M., Mahil S.K., Choon S.-E., Burden A.D., Young H.S., Carr I.M., Lachmann H.J., Lombardi G. (2020). IL-36 Promotes Systemic IFN-I Responses in Severe Forms of Psoriasis. J. Investig. Dermatol..

[197] Chandra A., Senapati S., Roy S., Chatterjee G., Chatterjee R. (2018). Epigenome-Wide DNA Methylation Regulates Cardinal Pathological Features of Psoriasis. Clin. Epigenetics.

[198] Chen M., Wang Y., Yao X., Li C., Jiang M., Cui P., Wang B. (2016). Hypermethylation of HLA-C May Be an Epigenetic Marker in Psoriasis. J. Dermatol. Sci..

[199] Zhou F., Shen C., Hsu Y.-H., Gao J., Dou J., Ko R., Zheng X., Sun L., Cui Y., Zhang X. (2018). DNA Methylation-Based Subclassification of Psoriasis in the Chinese Han Population. Front. Med..

[200] Li F., Yuan C.W., Xu S., Zu T., Woappi Y., Lee C.A.A., Abarzua P., Wells M., Ramsey M.R., Frank N.Y. (2020). Loss of the Epigenetic Mark 5-hmC in Psoriasis: Implications for Epidermal Stem Cell Dysregulation. J. Investig. Dermatol..

[201] Zeng C., Tsoi L.C., Gudjonsson J.E. (2021). Dysregulated Epigenetic Modifications in Psoriasis. Exp. Dermatol..

[202] Shao S., Gudjonsson J.E., Chang C., Lu Q. (2020). Epigenetics of Psoriasis. Epigenetics in Allergy and Autoimmunity.

[203] Dei-Cas I., Giliberto F., Luce L., Dopazo H., Penas-Steinhardt A. (2020). Metagenomic Analysis of Gut Microbiota in Non-Treated Plaque Psoriasis Patients Stratified by Disease Severity: Development of a New Psoriasis-Microbiome Index. Sci. Rep..

[204] Kim C.H. (2021). Control of Lymphocyte Functions by Gut Microbiota-Derived Short-Chain Fatty Acids. Cell. Mol. Immunol..

[205] Zhang X., Shi L., Sun T., Guo K., Geng S. (2021). Dysbiosis of Gut Microbiota and Its Correlation with Dysregulation of Cytokines in Psoriasis Patients. BMC Microbiol..

[206] Shapiro J., Cohen N.A., Shalev V., Uzan A., Koren O., Maharshak N. (2019). Psoriatic Patients Have a Distinct Structural and Functional Fecal Microbiota Compared with Controls. J. Dermatol..

[207] Schwarz A., Philippsen R., Schwarz T. (2021). Induction of Regulatory T Cells and Correction of Cytokine Disbalance by Short-Chain Fatty Acids: Implications for Psoriasis Therapy. J. Investig. Dermatol..

[208] Chang H.-W., Yan D., Singh R., Liu J., Lu X., Ucmak D., Lee K., Afifi L., Fadrosh D., Leech J. (2018). Alteration of the Cutaneous Microbiome in Psoriasis and Potential Role in Th17 Polarization. Microbiome.

[209] Fyhrquist N., Muirhead G., Prast-Nielsen S., Jeanmougin M., Olah P., Skoog T., Jules-Clement G., Feld M., Barrientos-Somarribas M., Sinkko H. (2019). Microbe-Host Interplay in Atopic Dermatitis and Psoriasis. Nat. Commun..

[210] Quan C., Chen X.-Y., Li X., Xue F., Chen L.-H., Liu N., Wang B., Wang L.-Q., Wang X.-P., Yang H. (2020). Psoriatic Lesions are Characterized by Higher Bacterial Load and Imbalance between *Cutibacterium* and *Corynebacterium*. J. Am. Acad. Dermatol..

[211] Chen L., Li J., Zhu W., Kuang Y., Liu T., Zhang W., Chen X., Peng C. (2020). Skin and Gut Microbiome in Psoriasis: Gaining Insight Into the Pathophysiology of It and Finding Novel Therapeutic Strategies. Front. Microbiol..

[212] Ng C.Y., Huang Y.H., Chu C.F., Wu T.C., Liu S.H. (2017). Risks for *Staphylococcus aureus* Colonization in Patients with Psoriasis: A Systematic Review and Meta-Analysis. Br. J. Dermatol..

[213] Abbas I., Al-Mohana A., Abbas E., Al-Dhalimi M. (2016). Cytokine Serum Level Association with Superantigen Production by *Staphylococcus aureus* in *Psoriasis vulgaris*. Res. J. Pharm. Biol. Chem. Sci..

[214] Polak K., Bergler-Czop B., Szczepanek M., Wojciechowska K., Frątczak A., Kiss N. (2021). Psoriasis and Gut Microbiome-Current State of Art. Int. J. Mol. Sci..

[215] Myers B., Brownstone N., Reddy V., Chan S., Thibodeaux Q., Truong A., Bhutani T., Chang H.-W., Liao W. (2019). The Gut Microbiome in Psoriasis and Psoriatic Arthritis. Best Pract. Res. Clin. Rheumatol..

[216] Chen Y.-J., Ho H.J., Tseng C.-H., Lai Z.-L., Shieh J.-J., Wu C.-Y. (2018). Intestinal Microbiota Profiling and Predicted Metabolic Dysregulation in Psoriasis Patients. Exp. Dermatol..

[217] Telfer N.R., Chalmers R.J.G., Whale K., Colman G. (1992). The Role of Streptococcal Infection in the Initiation of Guttate Psoriasis. Arch. Dermatol..

[218] Rachakonda T.D., Dhillon J.S., Florek A.G., Armstrong A.W. (2015). Effect of Tonsillectomy on Psoriasis: A Systematic Review. J. Am. Acad. Dermatol..

[219] Haapasalo K., Koskinen L.L.E., Suvilehto J., Jousilahti P., Wolin A., Suomela S., Trembath R., Barker J., Vuopio J., Kere J. (2018). The Psoriasis Risk Allele HLA-C*06:02 Shows Evidence of Association with Chronic or Recurrent Streptococcal Tonsillitis. Infect. Immun..

[220] Yong W.C., Upala S., Sanguankeo A. (2018). Association between Psoriasis and Helicobacter Pylori Infection: A Systematic Review and Meta-Analysis. Indian J. Dermatol..

[221] Pietrzak A., Grywalska E., Socha M., Roliński J., Franciszkiewicz-Pietrzak K., Rudnicka L., Rudzki M., Krasowska D. (2018). Prevalence and Possible Role of Candida Species in Patients with Psoriasis: A Systematic Review and Meta-Analysis. Mediat. Inflamm..

[222] Ahmed A.S., Al-Najjar A.H., Alshalahi H., Altowayan W.M., Elgharabawy R.M. (2021). Clinical Significance of Helicobacter Pylori Infection on Psoriasis Severity. J. Interferon Cytokine Res..

[223] De Jesús-Gil C., Sans-de San Nicolàs L., Ruiz-Romeu E., Ferran M., Soria-Martínez L., García-Jiménez I., Chiriac A., Casanova-Seuma J.M., Fernández-Armenteros J.M., Owens S. (2021). Interplay between Humoral and CLA(+) T Cell Response against *Candida albicans* in Psoriasis. Int. J. Mol. Sci..

[224] Camargo C.M.d.S., Brotas A.M., Ramos-e-Silva M., Carneiro S. (2013). Isomorphic Phenomenon of Koebner: Facts and Controversies. Clin. Dermatol..

[225] Zhang L.-J. (2019). Type1 Interferons Potential Initiating Factors Linking Skin Wounds with Psoriasis Pathogenesis. Front. Immunol..

[226] Ni X., Lai Y. (2020). Keratinocyte: A trigger or an Executor of Psoriasis?. J. Leukoc. Biol..

[227] Qiao P., Guo W., Ke Y., Fang H., Zhuang Y., Jiang M., Zhang J., Shen S., Qiao H., Dang E. (2019). Mechanical Stretch Exacerbates Psoriasis by Stimulating Keratinocyte Proliferation and Cytokine Production. J. Investig. Dermatol..

[228] Elewski B., Alexis A.F., Lebwohl M., Stein Gold L., Pariser D., Del Rosso J., Yosipovitch G. (2019). Itch: An Under-Recognized Problem in Psoriasis. J. Eur. Acad. Dermatol. Venereol..

[229] Kamiya K., Kishimoto M., Sugai J., Komine M., Ohtsuki M. (2019). Risk Factors for the Development of Psoriasis. Int. J. Mol. Sci..

[230] Nosbaum A., Dahel K., Goujon C., Nicolas J.-F., Mengeaud V., Vocanson M. (2021). Psoriasis is a Disease of the Entire Skin: Non-Lesional Skin Displays a Prepsoriasis Phenotype. Eur. J. Dermatol..

[231] Mack M.R., Kim B.S. (2018). The Itch–Scratch Cycle: A Neuroimmune Perspective. Trends Immunol..

[232] Dogra S., Kamat D. (2019). Drug-Induced Psoriasis. Indian J. Rheumatol..

[233] Shi C.R., Nambudiri V.E. (2017). Widespread Psoriasis Flare Following Influenza Vaccination. Vaccine.

[234] Choudhry A., Mathena J., Albano J.D., Yacovone M., Collins L. (2016). Safety Evaluation of Adenovirus Type 4 and Type 7 Vaccine Live, Oral in Military Recruits. Vaccine.

[235] Barrea L., Savanelli M.C., Di Somma C., Napolitano M., Megna M., Colao A., Savastano S. (2017). Vitamin D and Its Role in Psoriasis: An Overview of the Dermatologist and Nutritionist. Rev. Endocr. Metab. Disord..

[236] Brownstone N., Mosca M., Hong J., Hadeler E., Bhutani T. (2021). Phototherapy for Psoriasis: New Research and Insights. Curr. Dermatol. Rep..

[237] Sbidian E., Chaimani A., Garcia-Doval I., Doney L., Dressler C., Hua C., Hughes C., Naldi L., Afach S., Le Cleach L. (2021). Systemic Pharmacological Treatments for Chronic Plaque Psoriasis: A Network Meta-Analysis. Cochrane Database Syst. Rev..

[238] Nogueira M., Puig L., Torres T. (2020). JAK Inhibitors for Treatment of Psoriasis: Focus on Selective TYK2 Inhibitors. Drugs.

[239] Florek A.G., Wang C.J., Armstrong A.W. (2018). Treatment Preferences and Treatment Satisfaction among Psoriasis Patients: A Systematic Review. Arch. Dermatol. Res..

[240] Griffiths C.E.M., Armstrong A.W., Gudjonsson J.E., Barker J.N.W.N. (2021). Psoriasis. Lancet.

[241] Fluhr J.W., Cavallotti C., Berardesca E. (2008). Emollients, Moisturizers, and Keratolytic Agents in Psoriasis. Clin. Dermatol..

[242] Jacobi A., Mayer A., Augustin M. (2015). Keratolytics and Emollients and Their Role in the Therapy of Psoriasis: A Systematic Review. Dermatol. Ther..

[243] Maroto-Morales D., Montero-Vilchez T., Arias-Santiago S. (2021). Study of Skin Barrier Function in Psoriasis: The Impact of Emollients. Life.

[244] Pfaff C.M., Marquardt Y., Fietkau K., Baron J.M., Lüscher B. (2017). The Psoriasis-Associated IL-17A Induces and Cooperates with IL-36 Cytokines to Control Keratinocyte Differentiation and Function. Sci. Rep..

[245] Wang W., Yu X., Wu C., Jin H. (2017). IL-36γ Inhibits Differentiation and Induces Inflammation of Keratinocyte via Wnt Signaling Pathway in Psoriasis. Int. J. Med. Sci..

[246] Miura S., Garcet S., Salud-Gnilo C., Gonzalez J., Li X., Murai-Yamamura M., Yamamura K., Rambhia D., Kunjravia N., Guttman-Yassky E. (2021). IL-36 and IL-17A Cooperatively Induce a Psoriasis-Like Gene Expression Response in Human Keratinocytes. J. Investig. Dermatol..

[247] Bachelez H., Choon S.-E., Marrakchi S., Burden A.D., Tsai T.-F., Morita A., Turki H., Hall D.B., Shear M., Baum P. (2019). Inhibition of the Interleukin-36 Pathway for the Treatment of Generalized Pustular Psoriasis. N. Engl. J. Med..

[248] Choon S.E., Lebwohl M.G., Marrakchi S., Burden A.D., Tsai T.-F., Morita A., Navarini A.A., Zheng M., Xu J., Turki H. (2021). Study Protocol of the Global Effisayil 1 Phase II, Multicentre, Randomised, Double-Blind, Placebo-Controlled Trial of Spesolimab in Patients with Generalized Pustular Psoriasis Presenting with an Acute Flare. BMJ Open.

[249] Mrowietz U., Burden A.D., Pinter A., Reich K., Schäkel K., Baum P., Datsenko Y., Deng H., Padula S.J., Thoma C. (2021). Spesolimab, an Anti-Interleukin-36 Receptor Antibody, in Patients with Palmoplantar Pustulosis: Results of a Phase IIa, Multicenter, Double-Blind, Randomized, Placebo-Controlled Pilot Study. Dermatol. Ther..

[250] Tsai Y.-C., Tsai T.-F. (2017). Anti-Interleukin and Interleukin Therapies for Psoriasis: Current Evidence and Clinical Usefulness. Ther. Adv. Musculoskelet. Dis..

[251] Harden J.L., Johnson-Huang L.M., Chamian M.F., Lee E., Pearce T., Leonardi C.L., Haider A., Lowes M.A., Krueger J.G. (2015). Humanized Anti-IFN-γ (HuZAF) in the Treatment of Psoriasis. J. Allergy Clin. Immunol..

[252] Mease P.J., Gottlieb A.B., Berman A., Drescher E., Xing J., Wong R., Banerjee S. (2016). The Efficacy and Safety of Clazakizumab, an Anti–Interleukin-6 Monoclonal Antibody, in a Phase IIb Study of Adults with Active Psoriatic Arthritis. Arthritis Rheumatol..

[253] Furue K., Ito T., Tsuji G., Nakahara T., Furue M. (2020). The CCL20 and CCR6 Axis in Psoriasis. Scand. J. Immunol..

[254] Bouma G., Zamuner S., Hicks K., Want A., Oliveira J., Choudhury A., Brett S., Robertson D., Felton L., Norris V. (2017). CCL20 Neutralization by a Monoclonal Antibody in Healthy Subjects Selectively Inhibits Recruitment of CCR6(+) Cells in an Experimental Suction Blister. Br. J. Clin. Pharmacol..

[255] Laffan S.B., Thomson A.S., Mai S., Fishman C., Kambara T., Nistala K., Raymond J.T., Chen S., Ramani T., Pageon L. (2020). Immune Complex Disease in a Chronic Monkey Study with a Humanised, Therapeutic Antibody against CCL20 is Associated with Complement-Containing Drug Aggregates. PLoS ONE.

[256] Heidenreich R., Röcken M., Ghoreschi K. (2009). Angiogenesis Drives Psoriasis Pathogenesis. Int. J. Exp. Pathol..

[257] Luengas-Martinez A., Hardman-Smart J., Paus R., Young H.S. (2020). Vascular Endothelial Growth Factor-A as a Promising Therapeutic Target for the Management of Psoriasis. Exp. Dermatol..

[258] Hsu M.-C., Chen C.-C. (2016). Psoriasis Flare-Ups Following Sorafenib Therapy: A Rare Case. Dermatol. Sin..

[259] Adachi T., Hiraoka A., Okazaki H., Nagamatsu K., Izumoto H., Yoshino T., Tsuruta M., Aibiki T., Okudaira T., Yamago H. (2020). Exacerbation of *Psoriasis vulgaris* by Sorafenib Treatment for Hepatocellular Carcinoma. Clin. J. Gastroenterol..

[260] Ohashi T., Yamamoto T. (2019). Exacerbation of Psoriasis with Pustulation by Sorafenib in a Patient with Metastatic Hepatocellular Carcinoma. Indian J. Dermatol..

[261] Dumet C., Pottier J., Gouilleux V., Watier H. (2018). New Structural Formats of Therapeutic Antibodies for Rheumatology. Jt. Bone Spine.

[262] Papp K.A., Weinberg M.A., Morris A., Reich K. (2021). IL17A/F Nanobody Sonelokimab in Patients with Plaque Psoriasis: A Multicentre, Randomised, Placebo-Controlled, Phase 2b Study. Lancet.

[263] Membrive Jiménez C., Pérez Ramírez C., Sánchez Martín A., Vieira Maroun S., Arias Santiago S.A., Ramírez Tortosa M.D.C., Jiménez Morales A. (2021). Influence of Genetic Polymorphisms on Response to Biologics in Moderate-to-Severe Psoriasis. J. Pers. Med..

[264] Dand N., Duckworth M., Baudry D., Russell A., Curtis C.J., Lee S.H., Evans I., Mason K.J., Alsharqi A., Becher G. (2019). HLA-C*06:02 Genotype is a Predictive Biomarker of Biologic Treatment Response in Psoriasis. J. Allergy Clin. Immunol..

[265] Konrad R.J., Higgs R.E., Rodgers G.H., Ming W., Qian Y.-W., Bivi N., Mack J.K., Siegel R.W., Nickoloff B.J. (2019). Assessment and Clinical Relevance of Serum IL-19 Levels in Psoriasis and Atopic Dermatitis Using a Sensitive and Specific Novel Immunoassay. Sci. Rep..

[266] Chan S., Reddy V., Myers B., Thibodeaux Q., Brownstone N., Liao W. (2020). Machine Learning in Dermatology: Current Applications, Opportunities, and Limitations. Dermatol. Ther..

